# Attempting to Compensate for Reduced Neuronal Nitric Oxide Synthase Protein with Nitrate Supplementation Cannot Overcome Metabolic Dysfunction but Rather Has Detrimental Effects in Dystrophin-Deficient *mdx* Muscle

**DOI:** 10.1007/s13311-016-0494-7

**Published:** 2016-12-05

**Authors:** Cara A. Timpani, Adam J. Trewin, Vanesa Stojanovska, Ainsley Robinson, Craig A. Goodman, Kulmira Nurgali, Andrew C. Betik, Nigel Stepto, Alan Hayes, Glenn K. McConell, Emma Rybalka

**Affiliations:** 1 Centre for Chronic Disease, College of Health & Biomedicine, Victoria University, Melbourne, Victoria 8001 Australia; 2 Institute of Sport, Exercise & Active Living (ISEAL), Victoria University, Melbourne, Victoria 8001 Australia; 3 Australian Institute of Musculoskeletal Science (AIMSS), Western Health, Melbourne, Victoria 3021 Australia

**Keywords:** Duchenne muscular dystrophy, Nitrate supplementation, Metabolism, Glucose uptake, Mitochondria

## Abstract

Duchenne muscular dystrophy arises from the loss of dystrophin and is characterized by calcium dysregulation, muscular atrophy, and metabolic dysfunction. The secondary reduction of neuronal nitric oxide synthase (nNOS) from the sarcolemma reduces NO production and bioavailability. As NO modulates glucose uptake, metabolism, and mitochondrial bioenergetics, we investigated whether an 8-week nitrate supplementation regimen could overcome metabolic dysfunction in the *mdx* mouse. Dystrophin-positive control (C57BL/10) and dystrophin-deficient *mdx* mice were supplemented with sodium nitrate (85 mg/l) in drinking water. Following the supplementation period, extensor digitorum longus and soleus were excised and radioactive glucose uptake was measured at rest (basal) and during contraction. Gastrocnemius was excised and mitochondrial respiration was measured using the Oroboros Oxygraph. Tibialis anterior was analyzed immunohistochemically for the presence of dystrophin, nNOS, nitrotyrosine, IgG and CD45+ cells, and histologically to assess areas of damage and regeneration. Glucose uptake in the basal and contracting states was normal in unsupplemented *mdx *muscles but was reduced following nitrate supplementation in *mdx* muscles only. The mitochondrial utilization of substrates was also impaired in *mdx* gastrocnemius during phosphorylating and maximal uncoupled respiration, and nitrate could not improve respiration in *mdx* muscle. Although nitrate supplementation reduced mitochondrial hydrogen peroxide emission, it induced mitochondrial uncoupling in red gastrocnemius, increased muscle fiber peroxynitrite (nitrotyrosine), and promoted skeletal muscle damage. Our novel data suggest that despite lower nNOS protein expression and likely lower NO production in *mdx* muscle, enhancing NO production with nitrate supplementation in these mice has detrimental effects on skeletal muscle. This may have important relevance for those with DMD.

**Electronic supplementary material:**

The online version of this article (doi:10.1007/s13311-016-0494-7) contains supplementary material, which is available to authorized users.

## Introduction

Duchenne muscular dystrophy (DMD) is a progressive X-linked [1] neuromuscular disease affecting 1 in 3500 to 5000 live male births [2], which arises from the ablation of the cytoskeletal protein, dystrophin [3]. Dystrophin deficiency causes alterations to the myofiber architecture leading to membrane lesions, calcium (Ca^2+^) accumulation, muscular weakness, and cyclic bouts of degeneration and regeneration until the regenerative capacity of the muscle is unable to match demand for repair [4]. Damaged muscle is eventually replaced with fibrous and/or fatty connective tissue leading to a decrease in muscle function, with cardiorespiratory failure ensuing by the third decade of life [5].

Mitochondrial and metabolic dysfunction have been increasingly implicated in the pathogenesis of DMD, although it is not known if these abnormalities are associated with dystrophin deficiency, the pathophysiological sequelae caused by dystrophin deficiency, or completely independent of dystrophin deficiency [6]. Indeed, the only obvious physical link between dystrophin and the intracellular metabolic pathways is via neuronal nitric oxide synthase (nNOS), whereby ablation of dystrophin from the sarcolemma induces the secondary loss of the dystrophin-associated proteins [7], including nNOS [8, 9]. nNOS produces nitric oxide (NO), a key signaling molecule in skeletal muscle that regulates various biological processes, including blood flow, contraction, mass, satellite cell activation, Ca^2+^ handling, and glucose uptake (GU), in addition to mitochondrial metabolism, gene expression, and reactive oxygen species (ROS) production [10]. In dystrophic muscle, the dissociation of nNOS from the sarcolemma results in reduced nNOS content [11–14], activity [9, 15, 16] and NO production [17–19]. Importantly, this loss of nNOS has been shown to contribute to the progression of the dystrophic condition and to the deficits in metabolic function. For example, nNOS is a positive allosteric regulator of phosphofructokinase, the rate-limiting enzyme of the glycolytic pathway [20], and therefore plays a critical role in regulating glucose metabolism. Interestingly, DMD is not only associated with impairments in glycolysis [20–22], but also in β-fatty acid oxidation, the tricarboxylic acid cycle, and the electron transport system (ETS) (for detailed reviewed see [6]). Collectively, these metabolic impairments result in reduced energy production [23], with reports of adenosine triphosphate (ATP) content being 50% lower under resting conditions [24, 25]. Given that nNOS localization and NO signaling are known to be important for metabolic control, the loss of nNOS, and NO bioavailability might be key to metabolic deregulation in dystrophic skeletal muscle. Therefore, increasing NO availability has the potential to be of therapeutic benefit.

In an attempt to normalize NO production, several studies have reintroduced nNOS into dystrophic skeletal muscle which demonstrably reduces muscle damage and inflammation [26, 27]. As gene therapy for nNOS transfection is not yet available in humans, other strategies to restore NO availability have been investigated. Several studies have shown that supplementation with NO donors, often combined with anti-inflammatory drugs, results in reduced damage, necrosis and inflammation, and improved muscle blood flow, function/strength, and repair [28–35] in dystrophin-deficient skeletal muscle. While these findings may suggest a positive effect of increasing NO availability, it is difficult to control the delivery of NO to the skeletal muscle with pharmacological donors and also to separate the effects of the NO donor from those of the anti-inflammatory co-treatment. Another approach to increase NO availability has been to supplement with the nNOS substrate, ˪-arginine [19]; however, the potential for ˪-arginine to increase NO production is limited by the lowered nNOS protein in dystrophic skeletal muscle. An alternative method to increase NO availability, that is independent of nNOS activity, is supplementation with nitrate (NITR). Specifically, dietary NITR can be reduced to nitrite by commensal bacteria of the oral cavity and gastrointestinal tract, with nitrite being subsequently reduced to NO via several enzymatic pathways in the blood and tissues [36]. This mechanism is complementary to NOS-derived NO production and, importantly, represents a pathway that could be exploited to increase NO availability in dystrophic muscle.

To date, no studies have investigated the effect of NITR supplementation on metabolic function in dystrophic muscle; however, recent studies suggest that NITR supplementation has the potential to improve metabolic function in skeletal muscle. For example, Larsen et al. [37] demonstrated that NITR supplementation in healthy, young males led to increased plasma NO concentration and, subsequently, downstream metabolic adaptations, including increased mitochondrial efficiency, reduced proton leak, and ultimately increased ATP production capacity. Similar data have been derived in rats during fatty-acid oxidation [38]. In addition, there is some evidence that NITR supplementation can increase exercise efficiency in humans [39–41] and exercise capacity in some disease conditions such as peripheral arterial disease, where NO production is reduced [42]. Most pertinently, increasing NO bioavailability through administration of sodium nitrite mitigates functional ischemia in patients with Becker muscular dystrophy [43], suggesting that expansion of the NITR–nitrite–NO pool in DMD may also be beneficial. The results from these studies prompted us to investigate whether increasing NO availability via NITR supplementation, which has been previously proven to increase plasma [37, 44] and skeletal muscle [38] NO levels and elicit beneficial mitochondrial adaptations at the skeletal muscle level [37, 44], would improve mitochondrial function and rectify energy homeostasis dysregulation in dystrophic muscle. Therefore, we investigated whether an established dietary NITR supplementation regimen [44] could improve GU, mitochondrial function, ROS emission, and muscle architecture in healthy (control; CON) and dystrophic (*mdx*) mouse models. We hypothesized that NITR supplementation would 1) increase GU in the contracting muscles from CON and *mdx* mice; 2) improve mitochondrial function in *mdx* mice and; 3) improve the muscle architecture of *mdx* mice.

## Materials and Methods

### Ethical approval

All experimental procedures were approved by the Victoria University Animal Ethics Experimentation Committee and conformed to the Australian Code of Practice for the Care and Use of Animals for Scientific Purposes.

### Animals and Supplementation

Three-week-old male C57Bl/10ScSn (normal wild-type strain; CON) and C57Bl/10*mdx* (*mdx*) mice were purchased from Animal Resources Centre (Western Australia, Australia) and housed at the Western Centre for Health, Research and Education (Sunshine Hospital, Victoria, Australia) on a 12:12 h light:dark cycle with ad libitum access to food and water. Following a 1-week acclimatization period, mice were randomly assigned into 4 groups: unsupplemented (CON UNSUPP and *mdx* UNSUPP) and supplemented (CON NITR and *mdx* NITR). Mice in the supplemented groups were given 85 mg/l (1 mM) sodium NITR [44] ad libitum in drinking water for 8 weeks and mice in the unsupplemented groups were given drinking water without NITR. The dose of NITR is comparable with doses studied in human experiments, is achievable through a normal diet [45], and is proven to increase the plasma NITR–nitrite–NO pool [37, 44].

### Materials and Antibodies

All chemicals, unless stated otherwise, were purchased from Sigma-Aldrich Chemicals (St. Louis, MO, USA). 2-Deoxy-d-[1,2-^3^H]glucose and d-[^14^H]mannitol were purchased from Perkin Elmer (Waltham, MA, USA). Dystrophin (ab15277), nNOS (ab1376), nitrotyrosine (ab42789), and Total OXPHOS (ab110413) primary antibodies were purchased from Abcam (Cambridge, MA, USA). Secondary antibodies for immunohistochemistry were purchased from Jackson Immunoresearch Laboratories (West Grove, PA, USA) and for Western blotting from Vector Laboratories (Burlingame, CA, USA).

### Muscle Dissection and Contraction Protocol

Mice were deeply anesthetized via intraperitoneal injection of sodium pentobarbitone (60 mg/kg) and the white extensor digitorum longus (EDL) and red soleus (SOL) muscle proximal and distal tendons in each hindlimb were tied with 4/0 surgical silk. Both EDL and SOL were surgically dissected tendon-to-tendon and placed in individual muscle baths containing Krebs basal buffer [118.5 mM NaCl, 24.7 mM NaHCO_3_, 4.7 4 mM KCl, 1.18 mM MgSO_4_, 2.5 mM CaCl_2_, 8 mM mannitol, 2 mM Na pyruvate, 0.01% bovine serum albumin (BSA); pH 7.4] bubbled with carbogen (95% O_2_, 5% CO_2_) at 30 °C. The proximal tendon was attached to a force transducer and muscles were rested for 20 min to equilibrate in the bath. Muscles were stimulated via square-wave electrical pulses delivered by platinum electrodes flanking the muscles, and subsequent recording of the force output were obtained from a custom-built muscle analysis system (Zultek Engineering, Victoria, Australia). Following determination of optimal length (L_o_) for each muscle via a succession of isometric twitch contractions, the left EDL and SOL were stimulated to contract for a total of 10 min (pulse durations of 350 ms and 500 ms for EDL and SOL, respectively, at a frequency of 60 Hz). This protocol maintains muscle viability and maximizes GU [46]. The right EDL and SOL were not stimulated in order to measure basal GU.

### GU

Following 5 min of contraction, the Krebs basal buffer was exchanged for Krebs buffer with 2-deoxy-d-[1,2-^3^H]glucose (0.128 μCi/ml) and d-[^14^H]mannitol (0.083 μCi/ml) in both resting and contracting muscles. At the end of the 10-min contraction protocol, muscles were immediately submerged in ice-cold Krebs basal buffer to stop further glucose uptake, blotted on filter paper, and snap frozen in liquid nitrogen. Whole muscles were weighed frozen, digested for 10 min at 95 °C in 135 μl 1 M NaOH, neutralized with 135 μl 1 M HCl, and centrifuged for 5 min at 13,000 *g*. In total, 200 μl supernatant was added to 4 ml inorganic scintillation fluid (UltimaGold; Perkin Elmer) and radioactivity was measured in a β-scintillation counter (Tri-Carb 2810; Perkin Elmer). GU was calculated as previously described [47].

### Mitochondrial Respiration and Hydrogen Peroxide Emission Measurements

Left and right gastrocnemius were excised from the anesthetized mice, separated into red gastrocnemius (RG) and white gastrocnemius (WG) portions and immediately placed into ice-cold BIOPS [7.23 mM K_2_ ethylene glycol-bis(β-aminoethyl ether)-N,N,N’,N’-tetracetic acid (EGTA), 2.77 mM CaK_2_EGTA, 5.77 mM Na_2_ATP, 6.56 mM MgCl_2_–6H_2_O, 20 mM taurine, 15 mM phosphocreatine, 20 mM imidizole, 0.5 mM dithiothreitol, 50 mM K^+^–MES; pH 7.1]. Muscle fibers were mechanically separated from a small portion of muscle in ice-cold BIOPS to maximize fiber surface area and transferred into ice-cold BIOPS supplemented with saponin (50 μg/ml) for 30 min. Separated fibers were agitated to permeabilize the sarcolemma and allow diffusion of subsequent assay substrates, and then washed 3 times via agitation in ice-cold respiration buffer (110 mM K^+^–MES, 35 mM KCl, 1 mM EGTA, 5 mM K_2_HPO_4_, 3 mM MgCl_2_–6H_2_O, 0.05 mM pyruvate, 0.0 2 mM malate, 5 mg/ml BSA; pH 7.4). Fiber bundles were then divided and weighed on a microbalance (2–4 mg each) for subsequent respirometry analysis in duplicate.

ETS respiration, OXPHOS, and hydrogen peroxide (H_2_O_2_) emission were measured by the Oxygraph O2k high-resolution respirometer (Oroboros Instruments, Innsbruck, Austria) via a substrate, uncoupler, inhibitor titration (SUIT) protocol at 37 °C in MIR05 respiration medium while stirring at 750 rpm, as previously described [48, 49], with minor modifications in order to assess H_2_O_2_ emission. Briefly, after fibers and oxygen (O_2_) were added to the respiration chamber, the SUIT protocol commenced with titrations of the complex I (CI) substrates malate (2 mM final concentration) and pyruvate (10 mM), followed by the complex II (CII) substrate succinate (10 mM) to determine leak (state 4) respiration. Titrations of adenosine diphosphate (ADP; 0.25, 1, and 5 mM) assessed OXPHOS (state 3) capacity, addition of cytochrome *c* (10 μM) tested mitochondrial membrane integrity, and titrations of FCCP (0.025 μM) determined uncoupled respiration. Complex-specific respiration was inhibited by the addition of rotenone (1 μM) and antimycin A (5 μM) to CI and complex III (CIII), respectively. Finally, complex IV (CIV) capacity was measured during oxidation of TMPD (0.5 mM) with ascorbate (2 mM). The O_2_ flux due to auto-oxidation of these chemicals was determined after inhibition of CIV with sodium azide (75 mM) then subtracted from the raw CIV O_2_ flux.

Mitochondrial H_2_O_2_ emission was simultaneously measured in the respiration chamber throughout the SUIT protocol via optical sensors (O2k-Fluorescence LED-2 Module; Oroboros, Innsbruck, Austria) as previously described [50–52]. Superoxide (O_2_^–^) produced during the SUIT protocol was converted to H_2_O_2_ owing to the presence of a saturating concentration of O_2_^–^ dismutase (2.5 U/ml), and the subsequent H_2_O_2_ generation was quantified via the reaction of Amplex UltraRed (25 μM; Molecular Probes, Invitrogen, Carlsbad, CA, USA) with horseradish peroxidase (2.5 U/ml) at excitation/emission 565/600 nm wavelength. The H_2_O_2_ detection chemicals were added to the chambers containing MIR05 respiration medium at the beginning of the experiment, prior to the addition of the muscle fibers.

Chamber O_2_ concentration was maintained between 300 and 450 nmol/ml. Mass-specific O_2_ flux and H_2_O_2_ emission was determined from steady-state flux normalized to tissue wet weight and adjusted for instrumental background and residual O_2_ consumption. Respiratory control ratios were calculated (complex specific O_2_ flux relative to maximal uncoupled ETS respiration) to investigate intrinsic mitochondrial function independent of mitochondrial density.

### Western Blot Analysis of Mitochondrial Respiratory Chain Proteins

Frozen tissues were homogenized for 20 s in ice-cold WB buffer (40 mM Tris, pH 7.5; 1 mM ethylenediaminetetraacetic acid; 5 mM EGTA; 0.5% Triton X-100; 25 mM β-glycerophosphate; 25 mM NaF; 1 mM Na3VO4; 10 μg/ml leupeptin; and 1 mM phenylmethylsulfonyl fluoride), and the whole homogenate was used for further analysis. Sample protein concentrations were determined with a DC protein assay kit (Bio-Rad Laboratories, Hercules, CA, USA), and equivalent amounts of protein (15 μg) from each sample were dissolved in Laemmli buffer, heated for 5 min at 37 °C, and subjected to electrophoretic separation on sodium dodecyl sulfate polyacrylamide gel electrophoresis gels. Following electrophoretic separation, proteins were transferred to a polyvinylidene fluoride membrane, blocked with 5% powdered milk in Tris-buffered saline containing 0.1% Tween 20 (TBST) for 1 h followed by an overnight incubation at 4 °C with primary antibody dissolved in TBST containing 1% BSA. The Total OXPHOS Antibody Cocktail (1:1000), which detects representative proteins from each of the 5 mitochondrial respiratory chain complexes, was obtained from Abcam. This cocktail consisted of primary antibodies against the following proteins: NADH dehydrogenase (ubiquinone) 1 beta sub-complex, 8 (CI), succinate dehydrogenase assembly factor 4 (CII), ubiquinol-cytochrome-C reductase complex core protein 2 [complex III (CIII)], mitochondrially encoded cytochrome C oxidase I (CIV), and mitochondrial ATP synthase subunit alpha [complex V (CV)]. After overnight incubation, the membranes were washed for 30 min in TBST and then probed with a peroxidase-conjugated secondary antibody (1:10,000, antimouse; Vector Laboratories) for 1 h at room temperature. Following 30 min of washing in TBST, the blots were developed with a DARQ CCD camera mounted to a Fusion FX imaging system (Vilber Lourmat, Eberhardzell, Germany) using ECL Prime reagent (Amersham, Piscataway, NJ, USA). Once the images were captured, the membranes were stained with Coomassie Blue to verify equal loading of total protein in all lanes. Densitometric measurements were carried out using Fusion CAPT Advance software (Vilber Lourmat).

### Citrate synthase activity

Homogenized RG and WG samples were utilized to measure citrate synthase (CS) activity as a marker of mitochondrial density [53, 54]. Homogenized samples were added to reagent cocktail (100 mM Tris Buffer, 1 mM DTNB, 3 mM acetyl coenzyme A) and CS activity was measured spectrophotometrically (412 nm at 25 °C) for 5 min following the addition of 10 mM oxaloacetate as described previously [23]. CS activity was calculated using the extinction coefficient of 13.6 [55]. CS activity was normalized to whole muscle protein concentration.

### Histological Analysis

Following excision, the right tibilalis anterior (TA) was frozen in liquid nitrogen-cooled isopentane and optimal cutting temperature compound (Sakura Finetek, Alphen aan den Rijn, the Netherlands). Embedded TAs were cryosectioned (10 μm) at –20 °C using a Leica (CM1950) cryostat and mounted onto glass slides (Menzel-Glaser).

#### Dystrophin and nNOS immunolabeling

Slides were fixed using a Cytofix/Cytoperm Plus kit (BD Biosciences, San Jose, CA, USA) for 5 min. After incubation with blocking serum [0.1 M phosphate buffered saline (PBS), 0.1% triton, 10% fetal bovine serum] for 1 h at room temperature, sections were labeled with primary antibodies: rabbit antidystrophin (1:400) and goat anti-nNOS (1:200) overnight at room temperature. After washing 3 times with a 0.1 M PBS and 0.1% triton X solution, samples were incubated with secondary antibodies: donkey antirabbit Alexa 488 (1:200) and donkey antigoat Alexa 594 (1:200) for 2 h at room temperature. Tissues were washed 3 times with a 0.1 M PBS and 0.1% triton X solution and mounted with fluorescent mounting medium (DAKO, North Sydney, Australia). Confocal microscopy was performed on an Eclipse Ti confocal laser scanning system (Nikon, Tokyo, Japan). Fluorophores were visualized using a 488-nm excitation filter for Alexa 488 or fluorescein isothiocyanate and a 559-nm excitation filter for Alexa 594 or Rhodamine Red. Z-series images were acquired at a nominal thickness of 0.5 μm (512 × 512 pixels). The density of dystrophin and nNOS immunoreactivity in TA sections was measured from 8 randomly captured images (total area size 2 mm^2^) per animal at 20× magnification. All images were captured under identical acquisition exposure time conditions and calibrated to standardized minimum baseline fluorescence. Images were converted from red, green, and blue to grayscale 8-bit then to binary; changes in fluorescence from the baseline were measured using Image J software (NIH, Bethesda, MD, USA). The area of immunoreactivity was then expressed as a percentage of the total area examined. Quantitative analyses were conducted blindly.

#### Nitrotyrosine, CD45, and IgG immunolabeling

Slides were fixed with 4% formaldehyde for 30 min at room temperature. After incubation with blocking serum (0.1 M PBS, 0.1% triton, 10% donkey serum) for 1 h at room temperature, sections were then labeled with primary anti-S-nitrotyrosine (rabbit; 1:200; Millipore, Billerica, MA, USA), anti-CD45 (pan-leukocyte marker; rat; 1:200), and anti-IgG isotype control (hamster; 1:200) primary antibodies overnight at room temperature. After washing 3 times with a 0.1 M PBS and 0.1% triton X solution, samples were incubated with the appropriate secondary antibody [anti-rabbit Alexa Fluor 647, 1:100 (Abacus ALS, UK); anti-rat Alexa Fluor 488 (Jackson Immunoresearch Laboratories); anti-hamster Alexa Fluor 594 (Jackson Immunoresearch Laboratories)] for 2 h at room temperature. A pan-nuclei marker 4',6-diamidino-2-phenylindole (DAPI) was added to the tissue sections and incubated for 2 min at room temperature, tissues were washed 3 times with a 0.1 M PBS and 0.1% triton X solution and mounted with an anti-fade fluorescent mounting medium. Excitation wavelengths were set to 640 nm for Alexa 647, 406 nm for Alexa 405, 408.8 nm for Alexa Fluor 488, and 561.8 nm for Alexa Fluor 594. The confocal microscope was calibrated to standardize the minimum baseline fluorescence for imaging nitrotyrosine, CD45, and IgG immunoreactivity in the TA cross sections. At time of analysis all files were converted to thresholded 8-bit binary images using ImageJ software from 8 randomly captured images per animal. Images were analyzed through the “analyze particles” function, recording the counts (to determine the number of DAPI^-^positive nuclei) and relative nitrotyrosine expression recorded as percentage area fraction in arbitrary units. CD45+ and IgG immunoreactivity was recorded in arbitrary units. Green pseudocolor images of nitrotyrosine (Alexa Fluor 647; magenta) were generated using ImageJ software for publication only.

#### Hematoxylin and eosin staining

Slides were air-dried and stained using a hematoxylin and eosin staining protocol, including a 30-s incubation in hematoxylin and a 1-min and 45-s incubation in eosin. Slides were imaged using a Zeiss Axio Imager Z2 microscope at 20× magnification. Fiber size, damaged area (areas of myofibril demise and inflammatory cell infiltration [56]), and fibers with centralized nuclei were determined using ImageJ software.

### Statistics

Results are presented as mean ± SEM. For all data, except for GU, a 2-way analysis of variance was utilized to detect between strain/genotype (CON vs. *mdx*) and supplementation (UNSUPP vs. NITR) differences. For GU data, a 3-way analysis of variance was performed for each of EDL and SOL to detect between strain, supplementation, and GU type (basal *vs* contraction). When a main effect or an interaction was detected, unpaired *t* tests were used to determine differences between individual groups using SPSS (version 21; IBM, Armonk, NY, USA). An α value of 0.05 was considered significant.

## Results

### Effect of NITR Supplementation on Body Weight, Food and Water Consumption, and Muscle Weights

**Fig. 1:**
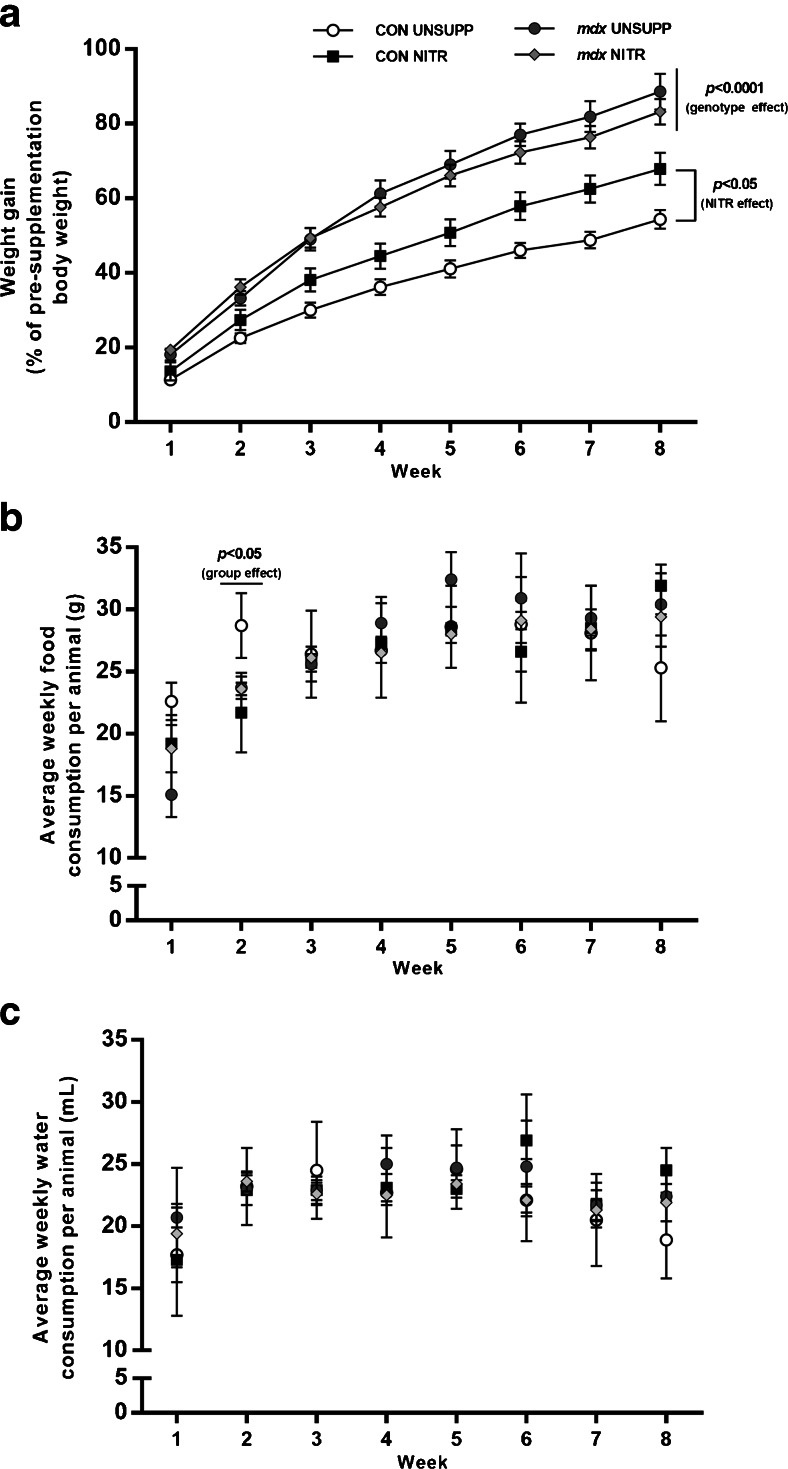
Body weight and average food and water consumption of unsupplemented (UNSUPP) and nitrate (NITR)-supplemented mice over the supplementation period. (**a**) Changes in body weight are shown as a percentage of presupplementation weight. Overall, *mdx* mice gained more weight over the 8-week supplementation period than control mice (CON; *p* < 0.0001). NITR had no effect on *mdx* weight gain but did increase weight gain in CON compared with CON UNSUPP (*p* < 0.05). Over the 8-week supplementation period, (**b**) food and (**c**) water consumption did not differ between unsupplemented and supplemented animals (*p* > 0.05) except for food consumption during week 2, where CON UNSUPP consumed more than all groups (*p* < 0.05). CON UNSUPP *n* = 16; CON NITR *n* = 17; *mdx* UNSUPP *n* = 14; *mdx* NITR *n* = 18

Throughout the 8-week supplementation period, greater weight gains were observed in the *mdx* groups compared with CON (*p* < 0.0001; Fig. 1a), with NITR having no effect in *mdx* mice (*p* > 0.05). NITR did, however, stimulate weight gain in CON (*p* < 0.05). No significant difference in food or water consumption was observed between any group over the supplementation period (Fig. 1b, c, respectively; *p* > 0.05) except at week 2, where food consumption was greater in CON UNSUPP than in all groups (*p* < 0.05; Fig. 1b). Overall, individual hindlimb muscle weights were greater in the *mdx* than in the CON strain (*p* < 0.0001; Tables 1 and 2) with NITR having no effect (*p* > 0.05).

**Table 1:** Weights of extensor digitorum longus (EDL) and soleus (SOL) used in glucose uptake experimentation*

	Left EDL (mg)	Right EDL (mg)	Left SOL (mg)	Right SOL (mg)
CON UNSUPP (*n* = 14)	12.3 ± 0.8	12.4 ± 1.2	9.0 ± 0.9	9.8 ± 0.9
CON NITR (*n* = 16)	11.3 ± 0.3	12.6 ± 0.8	10.3 ± 0.5	10.3 ± 0.6
*mdx* UNSUPP (*n* = 12)	14.8 ± 0.8^†^	15.5 ± 1.4^†^	13.5 ± 1.3^†^	14.9 ± 1.4^†^
*mdx* NITR (*n* = 13)	14.7 ± 0.9^†^	16.0 ± 0.8^†^	13.7 ± 0.8^†^	13.3 ± 0.6^†^

*Irrespective of supplementation, both EDL and SOL weights were significantly higher in *mdx* mice compared with control (CON) mice. There was no effect of nitrate (NITR)

^†^Significant difference from CON mice (*p* < 0.0001)

**Table 2:** Weights of the left gastrocnemius used in mitochondrial respiration experimentation, determination of citrate synthase activity and Western blotting of mitochondrial complexes and the right tibilalis anterior (TA) used for immunohistochemistry*

	Gastrocnemius (mg)	TA (mg)
CON UNSUPP (*n* = 13)	145.18 ± 4.4	46.9 ± 1.7
CON NITR (*n* = 16)	145.2 ± 3.3	46.5 ± 1.5
*mdx* UNSUPP (*n* = 12)	169.9 ± 4.2^†^	68.9 ± 2.1^†^
*mdx* NITR (*n* = 13)	170.2 ± 4.5^†^	66.9 ± 4.3^†^

*Irrespective of supplementation, gastrocnemius was significantly higher in *mdx* mice compared with control (CON) mice. There was no effect of nitrate (NITR). Similarly, TA was significantly higher in *mdx* mice compared with CON mice with no effect of NITR observed in either strain

^†^Significant difference from CON mice (*p* < 0.0001)

### Immunolabeling of Dystrophin and nNOS

To confirm the deficiency of both dystrophin and nNOS in *mdx* skeletal muscle, the presence of dystrophin and nNOS protein was determined in the TA (Fig. S1). Indeed, dystrophin was only evident in CON TA (Fig. S1A, C) and was absent from *mdx* TA except for a few spontaneously revertant fibers (*p* < 0.0001). Similarly, nNOS was only evident in CON TA (Fig. S1A^I^, C^I^), and was completely absent from dystrophin-deficient *mdx* fibers (*p* < 0.0001). Co-localization of dystrophin and nNOS was only observed in CON (Fig. S1A^II^, C^II^), and NITR had no effect on either dystrophin or nNOS expression (*p* > 0.05).

### Effect of NITR Supplementation on GU

**Fig. 2:**
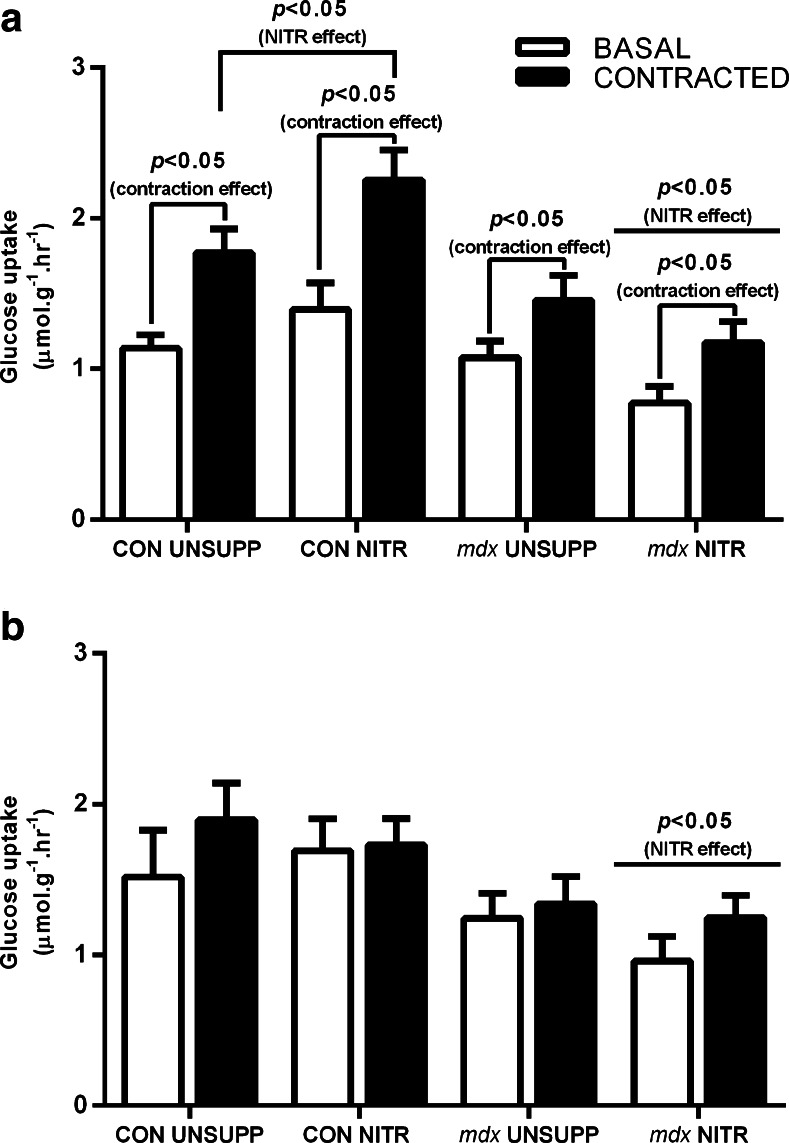
Glucose uptake (GU) in isolated extensor digitorum longus (EDL) and soleus (SOL) from unsupplemented (UNSUPP) and nitrate (NITR) supplemented CON and *mdx* mice. (**a**) In all groups, contraction-induced GU significantly increased compared with basal conditions (*p* < 0.05). NITR increased contraction-induced GU in CON EDL (*p* < 0.05) but, in contrast, reduced both basal- and contraction-induced GU in *mdx* EDL (*p* < 0.05). (**b**) For the SOL, both basal- and contraction-induced GU were comparable (*p* > 0.05). NITR reduced basal GU in *mdx* SOL (*p* < 0.05) but had no effect in CON SOL. CON UNSUPP *n* = 9–13; CON NITR *n* = 11; *mdx* UNSUPP *n* = 11; *mdx* NITR *n* = 10–12

NO has been proposed to play a role in contraction-stimulated GU and, as such, we first investigated the effect of NITR supplementation on GU in CON and *mdx* muscles. This is the first instance of contraction-induced GU being measured in the *mdx* mouse and we demonstrated no difference in basal- or contraction-induced GU between CON and *mdx* UNSUPP EDL (*p* > 0.05; Fig. 2a). As expected, contraction induced an increase in GU in the EDLs of CON UNSUPP (55%), CON NITR (61%), *mdx* UNSUPP (35%), and *mdx* NITR (51%) compared with basal conditions (*p* < 0.05; Fig. 2a). NITR supplementation significantly increased contraction-induced GU in CON EDL muscles (*p* < 0.05; Fig. 2a); however, in contrast, NITR reduced both basal- and contraction-induced GU in *mdx* muscles (*p* < 0.05; Fig. 2a). Contrary to the EDL, contraction did not stimulate further GU beyond that observed in basal conditions for any group in the SOL (all < 20%, *p* > 0.05; Fig. 2b). While NITR had no effect on basal or contraction-induced GU in CON SOL muscles (*p* > 0.05; Fig. 2b), NITR further reduced both basal and contraction-induced GU in *mdx* SOL muscles (*p* < 0.05)*.* Combined, these data suggest that NITR supplementation has a negative effect on GU in both *mdx* EDL and SOL, which may lead to impairments in downstream glycolysis and oxidative metabolism.

### Effect of NITR Supplementation on Mitochondrial Function

#### Respirometry

**Fig. 3:**
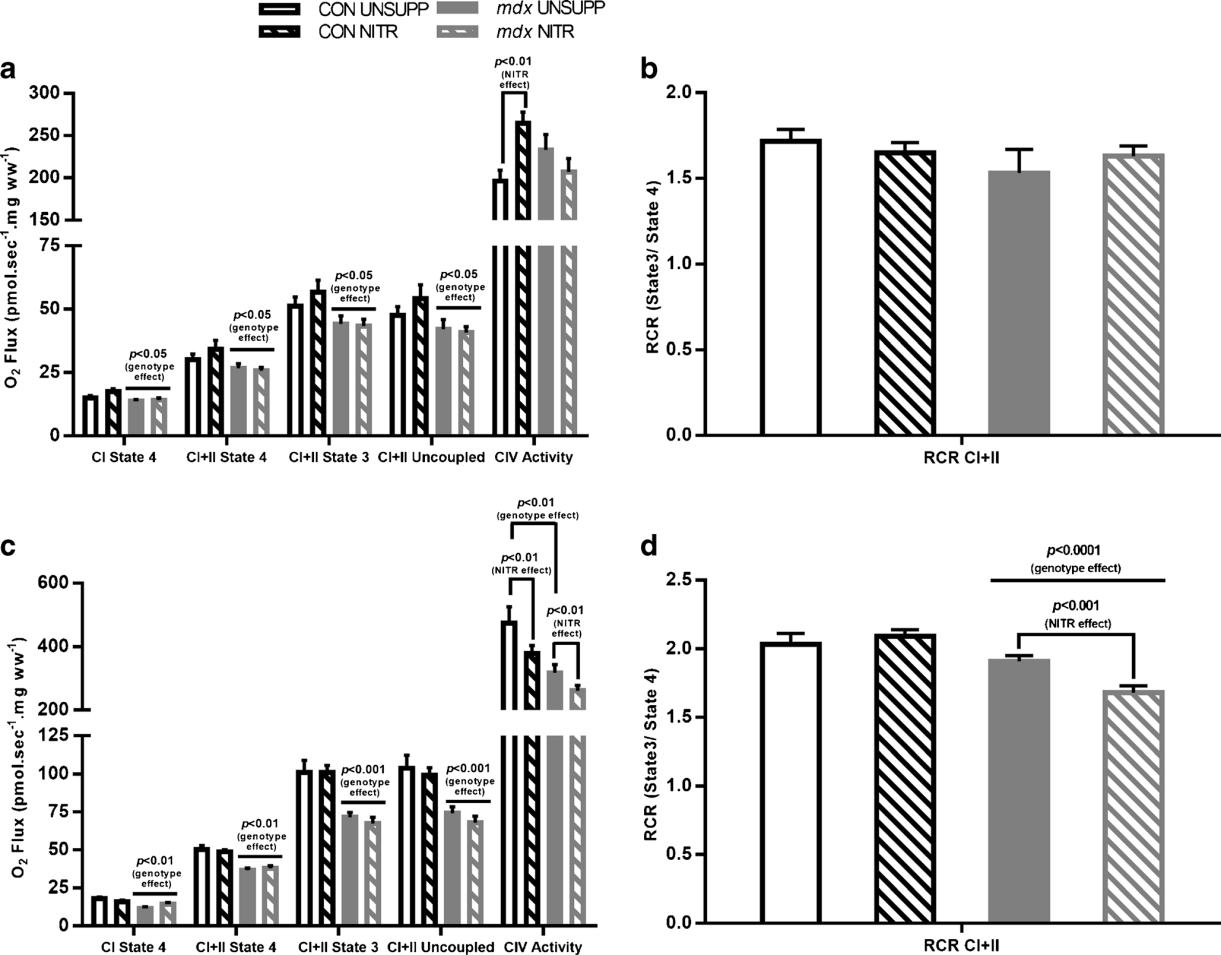
Mitochondrial function in intact, permeabilized muscle fibers from the white (WG) and red portion of gastrocnemius (RG) from unsupplemented (UNSUPP) and nitrate (NITR)-supplemented control (CON) and *mdx* mice. (**a**) State 4 leak respiration is significantly reduced in *mdx* compared with CON WG, irrespective of substrate combination (*p* < 0.05). (**a**) Adenosine diphosphate-stimulated state 3 respiration is significantly reduced in *mdx* compared with CON WG (*p* < 0.05) with NITR having no effect. (**a**) FCCP-stimulated uncoupled respiration is significantly reduced in *mdx* WG compared with CON (*p* < 0.05) with no difference in complex IV (CIV) activity detected between CON and *mdx* WG (*p* > 0.05). (**b**) The respiratory control ratio (RCR), an indicator of the coupling of O_2_ consumption and adenosine triphosphate production at the electron transport system, was comparable between CON and *mdx* WG, although a trend for *mdx* to be lower was detected (*p* = 0.083). When respiring on complex I (CI) + complex II (CII) substrates, the RCR was significantly lower across all groups (*p* < 0.0001). (**c**) State 4 leak respiration was significantly reduced in *mdx* compared with CON RG, irrespective of substrate combination (*p* < 0.01). ADP-stimulated state 3 respiration was significantly reduced in *mdx* compared with CON RG (*p* < 0.001) with NITR having no effect on phosphorylating respiration. FCCP-stimulated uncoupled respiration was significantly reduced in *mdx* compared with CON (*p* < 0.001). CIV activity was significantly reduced in *mdx* UNSUPP compared with CON UNSUPP (*p* < 0.01) with NITR inducing a significant decrease in both CON and *mdx* (*p* < 0.01). (**d**) The RCR in *mdx* RG during CI + II-stimulated respiration was lower compared with CON (*p* < 0.0001) with NITR decreasing the RCR in *mdx* during CI + II-stimulated respiration (*p* < 0.001, respectively). CON UNSUPP *n* = 12–13; CON NITR *n* = 12–13; *mdx* UNSUPP *n* = 10–11; *mdx* NITR *n* = 11–12

Next, we examined the effect of NITR on parameters of mitochondrial function. First, we measured state 4 leak respiration, which, in the absence of ADP, indicates the contribution of proton leak to respiration. In the presence of pyruvate and malate (CI), state 4 leak respiration was significantly lower in *mdx* WG (*p* < 0.05; Fig. 3a) and RG (*p* < 0.01; Fig. 3c) muscles compared with their respective controls. In the presence of pyruvate, malate, and succinate (CI + II), state 4 leak respiration was significantly higher than CI respiration across all groups, in both WG and RG muscles (*p* < 0.0001; Fig. 3a, c, respectively). NITR supplementation had no effect on either CI or CI + II state 4 leak respiration in CON or *mdx* muscles (Fig. 3a, c).

Next, the effect of NITR on coupled OXPHOS capacity was examined in WG and RG muscles by assessing maximal ADP-stimulated state 3 respiration in the presence of excess malate, pyruvate, and succinate (CI + II substrates). As shown in Fig. 3, state 3 respiration was significantly depressed in *mdx* WG by ~15% (*p* < 0.05; Fig. 3a) and in *mdx* RG by 25% (*p* < 0.001; Fig. 3c) compared with CON. NITR supplementation, however, had no effect on state 3 respiration in either muscle (Fig. 3a, c).

Maximal ETS capacity was then assessed by the addition of the uncoupling agent FCCP, which dissipates the mitochondrial membrane potential (ΔΨ). This parameter gives an indication of the maximal respiration in the uncoupled state. FCCP-induced maximal uncoupled respiration was significantly lower in *mdx* WG (*p* < 0.05; Fig. 3a) and RG (*p* < 0.001; Fig. 3c) compared with their respective controls; however, there was no effect of NITR on this parameter.

Next, we measured the activity of CIV (cytochrome C oxidase), the terminal oxidase of the ETS and the site of O_2_ reduction to water. As shown in Fig. 3, CIV activity was not different between UNSUPP CON and *mdx* WG muscles (Fig. 3a); however, in the RG muscles, CIV activity was significantly lower in *mdx* UNSUPP compared with CON UNSUPP (*p* < 0.01; Fig. 3c). NITR induced a significant increase in CIV activity in CON WG muscles (*p* < 0.01; Fig. 3a) but reduced CIV activity in both CON and *mdx* RG muscles (*p* < 0.01; Fig. 3c).

Finally, the respiratory control ratio (RCR; state 3 respiration divided by state 4 respiration) was calculated. The RCR is an indicator of the extent to which O_2_ consumption is coupled to ATP production and therefore mitochondrial efficiency, with a higher RCR indicating better coupling. No difference in RCR was observed between CON and *mdx* WG (*p* > 0.05; see Fig. 5b). In *mdx* RG respiring on CI + II, the RCR was significantly lower compared to CON (*p* < 0.0001) and NITR decreased the RCR further (*p* < 0.01; Fig. 3d). This highlights that in oxidative red muscle at least, *mdx* mitochondria are more uncoupled and that this uncoupling is exacerbated by NITR.

#### Electron transport chain complex expression

**Fig. 4:**
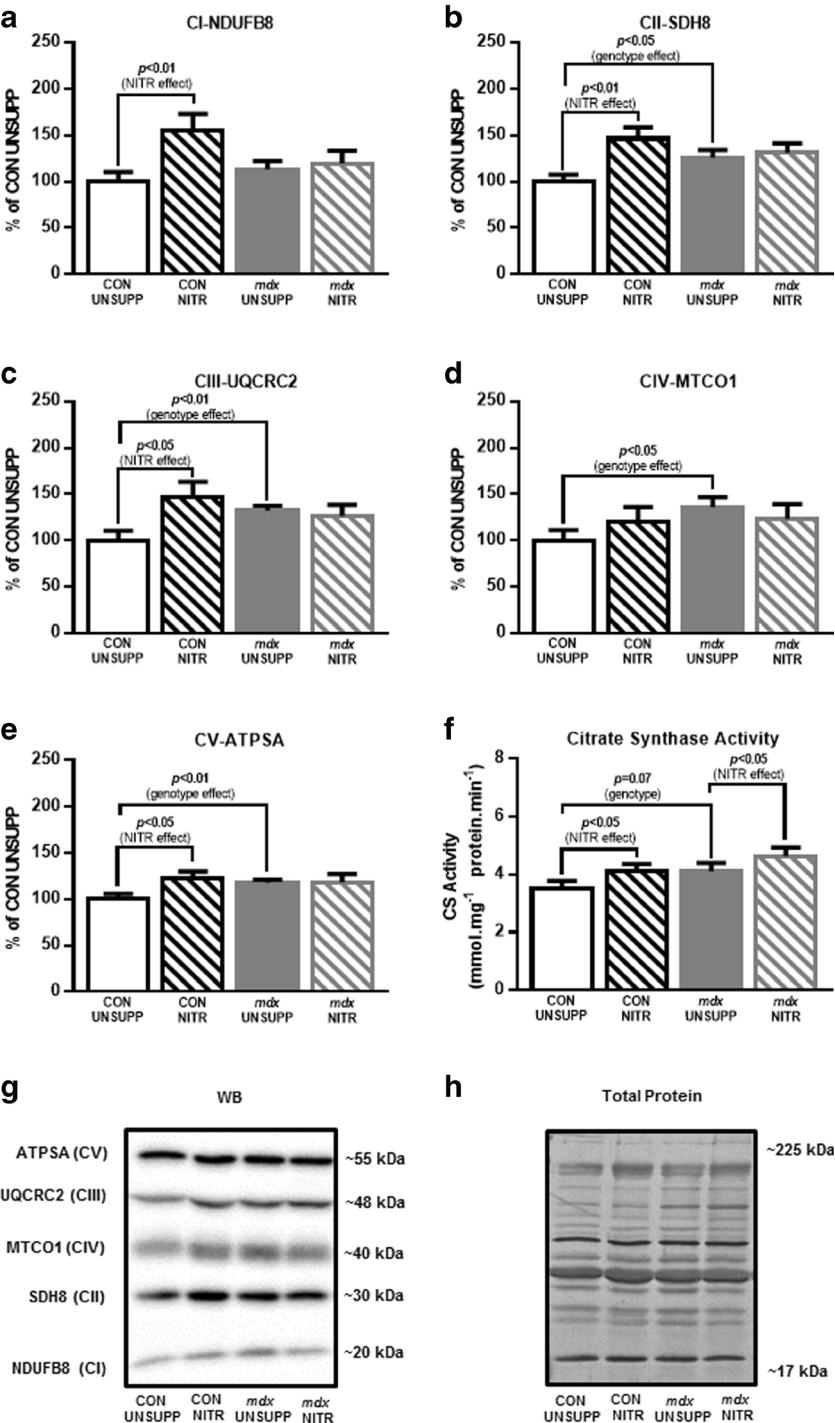
Mitochondrial respiratory chain complex proteins, and citrate synthase (CS) activity, from the white portion of gastrocnemius (WG) from unsupplemented (UNSUPP) and nitrate (NITR)-supplemented control (CON) and *mdx* mice. In *mdx* UNSUPP WG, expression of (**b**) complex II (CII; *p* < 0.05), (**c**) complex III (CIII; *p* < 0.01), (**d**) complex IV (CIV; *p* < 0.05), and (E) complex V (CV; *p* < 0.01) were greater compared with CON UNSUPP. NITR induced an increase in (**a**) CI (*p* < 0.01), (**b**) CII (*p* < 0.01), (**c**) CIII (*p* < 0.05), and (**e**) CV (*p* < 0.05) subunits in CON WG but not in *mdx* WG. (**f**) NITR also increased CS activity in both CON and *mdx* WG (*p* < 0.05) with a trend for CS activity to be higher in *mdx* UNSUPP compared with CON (*p* = 0.07). (**g**, **h**) Representative Western blots of proteins from each of the 5 mitochondrial respiratory complexes with Coomassie blue stains of the respective Western blots to demonstrate equal loading of the total protein. *n* = 8 per group

**Fig. 5:**
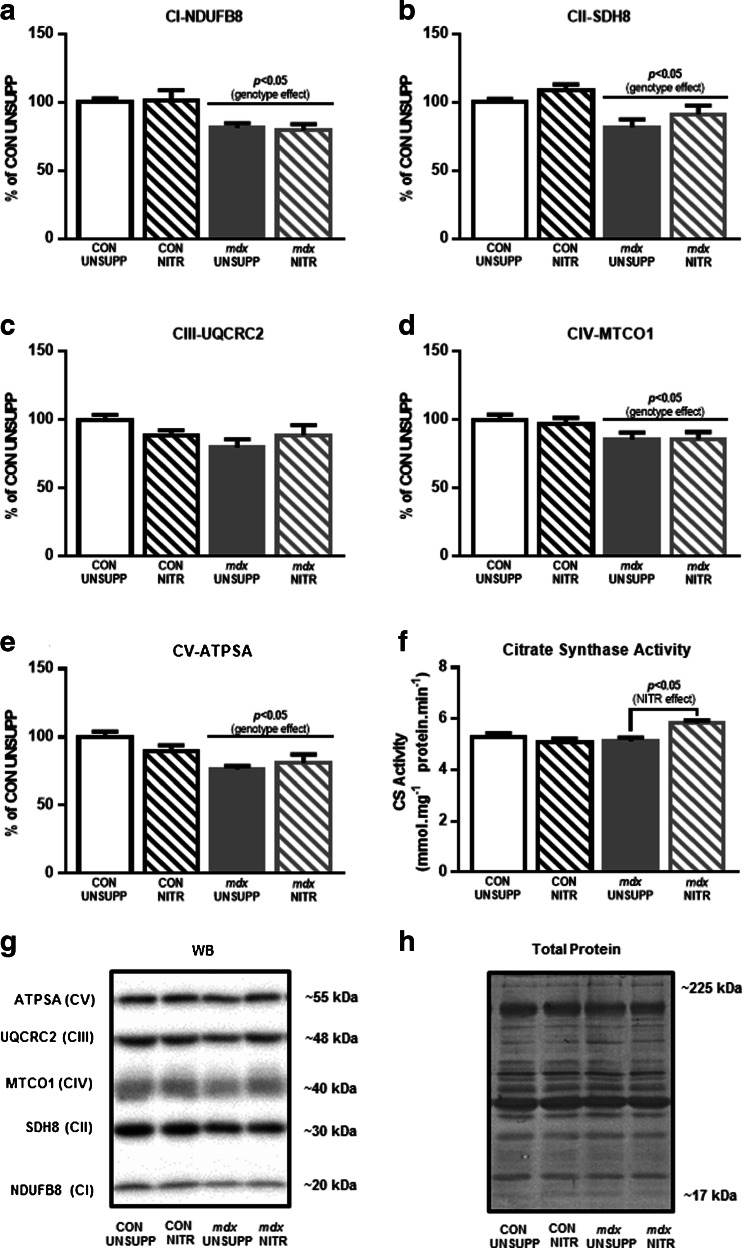
Mitochondrial respiratory chain complex proteins, and citrate synthase (CS) activity from the red portion of gastrocnemius (RG) from unsupplemented (UNSUPP) and nitrate (NITR) supplemented control (CON) and *mdx* mice. Overall, expression of (**a**) complex I (CI), (**c**) complex III (CIII), (**d**) complex IV (CIV), and (**e**) complex V (CV) subunits were decreased in *mdx* RG compared with CON (*p* < 0.05). (**f**) NITR increased CS activity in *mdx* RG but not in CON (*p* < 0.05). (**g**, **h**) Representative Western blots of proteins from each of the 5 mitochondrial respiratory complexes with Coomassie blue stains of the respective western blots to demonstrate equal loading of the total protein. *n* = 8 per group

To determine whether the genotypic differences and NITR supplementation-induced changes in respiration parameters were associated with differences in mitochondrial ETS complex densities, the abundance of representative proteins from each of the 5 ETS complexes were measured using semiquantitative Western blotting (Figs. 4 and 5). In WG muscles, despite state 3, state 4, and maximal uncoupled respiration being lower in *mdx* muscles (Fig. 3a), the relative abundance of representative proteins from complexes I to V were not lower. In fact, to the contrary, proteins from CII, CIII, CIV, and CV were significantly elevated in *mdx* UNSUPP WG muscles compared with CON UNSUPP muscles (Fig. 4). Interestingly, the NITR-induced increase in CIV respiratory activity (Fig. 3a) was not associated with a significant increase in the abundance of the CIV protein (Fig. 4d). NITR supplementation did, however, lead to an increase in representative proteins in WG muscles for CI, CII, CIII, and CV in CON but not *mdx* muscles (Fig. 4).

Unlike the WG muscles, the lower state 3, state 4, uncoupled respiration, and CIV activity found in RG *mdx* muscles (Fig. 3c) was accompanied by a reduction in representative proteins for CI, CII, CIV, and CV compared with CON; however, NITR supplementation had no effect on any of these proteins in either CON or *mdx* RG muscles (Fig. 5).

#### CS activity

Finally, we measured CS activity in WG and RG muscles as a co-marker of mitochondrial content alongside mitochondrial ETC proteins [53] (Figs. 4f and 5f, respectively). As shown in Fig. 4f, there was a trend for CS activity to be higher in *mdx* UNSUPP compared with CON UNSUPP WG muscles. Moreover, NITR increased CS activity in both CON and *mdx* WG muscles. In the RG muscles there was no difference in CS activity between UNSUPP CON and *mdx* mice; however, NITR increased CS activity in RG muscles from *mdx* mice. Overall, NITR did not improve the capacity to phosphorylate ATP or maximal respiratory capacity in dystrophic muscle despite increasing CS activity, suggesting that NITR may have an alternative effect on mitochondrial function such as ROS generation.

### Effect of NITR Supplementation on ROS Production in RG and WG

**Fig. 6:**
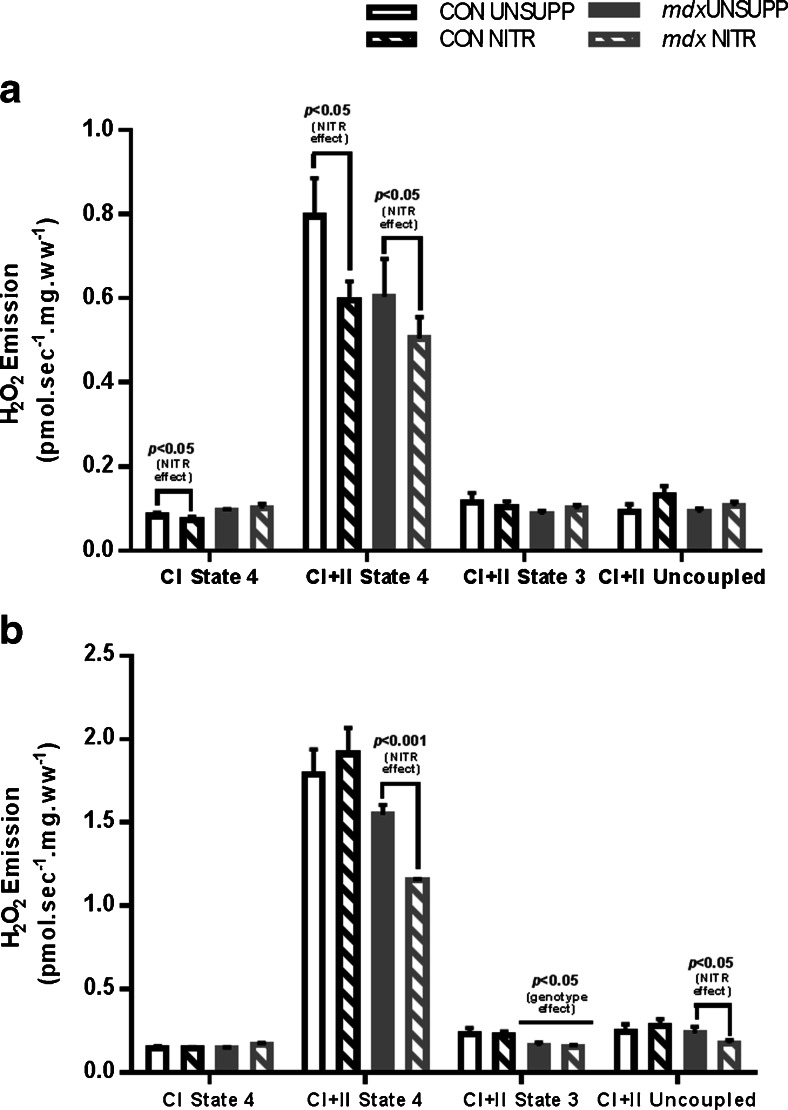
H_2_O_2_ emission in intact fibers from the (**a**) white (WG) and (**b**) red (RG) portions of gastrocnemius from unsupplemented (UNSUPP) and nitrate (NITR)-supplemented control (CON) and *mdx* mice. (**a**) In WG, NITR induced a decreased H_2_O_2_ emission during state 4 leak respiration in CON during complex I (CI)-stimulated respiration and in both CON and *mdx* during CI + complex II (CII)-stimulated respiration (*p* < 0.05). (**a**) In WG, no significant difference was detected in H_2_O_2_ emission during adenosine diphosphate (ADP)-stimulated state 3 respiration. There was no significant difference in H_2_O_2_ emission during FCCP-stimulated uncoupled respiration in WG. (**b**) In RG, NITR induced a decrease in H_2_O_2_ emission during state 4 leak respiration in *mdx* muscle during CI + CII-stimulated respiration (*p* < 0.001). (**b**) In *mdx* RG, H_2_O_2_ emission during ADP-stimulated state 3 respiration was significantly less compared with CON WG (*p* < 0.05) with NITR having no effect (*p* > 0.05). (**b**) While there were no differences in H_2_O_2_ emission during FCCP respiration between CON UNSUPP and *mdx* UNSUPP RG (*p* > 0.05), NITR reduced H_2_O_2_ emission in *mdx* RG compared with *mdx* UNSUPP (*p* < 0.05). CON UNSUPP *n* = 12–13; CON NITR *n* = 12–13; *mdx* UNSUPP *n* = 10–11; *mdx* NITR *n* = 11–12

The effect of NITR supplementation on the production of the mitochondrial ROS superoxide (O_2_^–^) was measured in intact and permeabilized fibers from WG and RG simultaneously with respiration. In the presence of excess O_2_^–^ dismutase, O_2_^–^ is converted to hydrogen peroxide (H_2_O_2_), which reacts with Amplex Red to produce the red fluorescent product, resorufin. During state 3 respiration, no differences in H_2_O_2_ emission were detected between CON and *mdx* UNSUPP WG (*p* > 0.05; Fig. 6a) with NITR having no effect in either strain (*p* > 0.05; Fig. 6a). NITR did, however, induce a decrease in H_2_O_2_ emission during state 4 leak respiration in CON WG muscle fibers respiring on CI substrates (*p* < 0.05; Fig. 6a). When respiring on CI + CII substrates, there was significantly greater H_2_O_2_ emission in all groups during state 4 leak respiration compared with CI substrates only in WG fibers (*p* < 0.0001; Fig. 6a). Importantly, NITR significantly decreased H_2_O_2_ emission in both CON and *mdx* WG muscles respiring during state 4 while on CI + II substrates (*p* < 0.05; Fig. 6a). There was no difference in H_2_O_2_ emission in WG between any groups during FCCP-stimulated maximal uncoupled respiration (*p* > 0.05; Fig. 6a).

In *mdx* RG fibers, there was significantly less H_2_O_2_ emission during state 3 respiration (*p* < 0.05; Fig. 6b) compared with CON fibers; however, there was no effect of NITR on this parameter (*p* > 0.05). Similar to WG fibers, H_2_O_2_ emission was higher when respiring on CI + CII substrates compared with CI substrates across all groups during state 4 leak respiration (*p* < 0.0001); however, NITR only reduced H_2_O_2_ emission in *mdx* fibers (*p* < 0 .001; Fig. 6b). NITR also reduced H_2_O_2_ emission in *mdx* RG fibers during FCCP uncoupled respiration (*p* < 0.05; Fig. 6b). While our data suggest that NITR reduces mitochondrial ROS production in dystrophic muscle, it is possible that increased NO bioavailability may sequester O_2_^–^ from the O_2_^–^ dismutase reaction to increase reactive nitrogen species (RNS).

### Effect of NITR Supplementation on Peroxynitrite Production, CD45^+^ Infiltration and IgG Immunolabeling

**Fig. 7:**
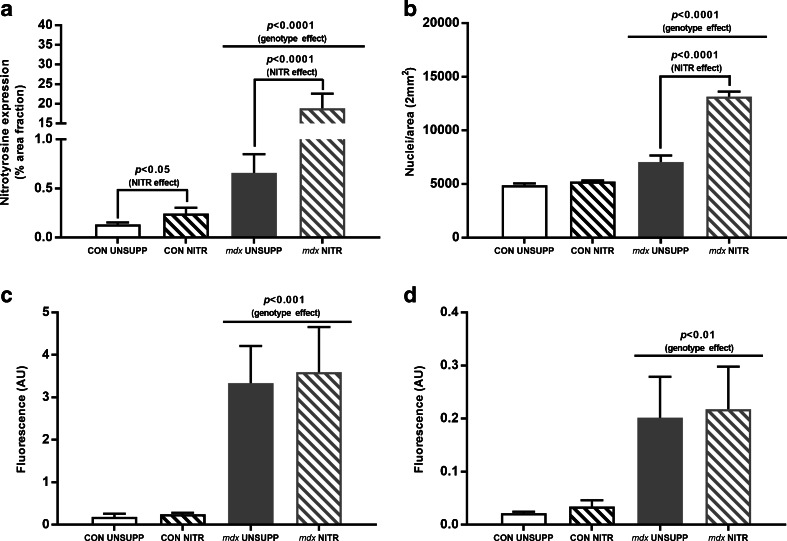
Immunohistological analysis of tibilalis anterior (TA) from unsupplemented (UNSUPP) and nitrate (NITR)-supplemented control (CON) and *mdx* mice. (**a**) Nitrotyrosine expression was higher in *mdx* TA compared with CON (*p* < 0.0001) with NITR supplementation elevating nitrotyrosine expression in both CON (*p* < 0.05) and *mdx* (*p* < 0.0001). (**b**) Nuclei content was higher in *mdx* TA compared with CON (*p* < 0.0001) with NITR further increasing nuclei content in *mdx* TA (*p* < 0.0001). (**c**) CD45 infiltration and (**d**) IgG staining was elevated in *mdx* TA compared with CON (*p* < 0.001 and *p* < 0.01, respectively) and NITR supplementation had no effect in either CON or *mdx* TA (*p* > 0.05). *Scale bars* = 100 μm. CON UNSUPP *n* = 3–4; CON NITR *n* = 3–4; *mdx* UNSUPP *n* = 3–4; *mdx* NITR *n* = 3–4

NO is known to react rapidly with O_2_^–^ resulting in the production of the highly RNS, peroxynitrite (ONOO^–^), and given that elevated ROS is present in *mdx* muscle [57], we investigated whether ONOO^–^ production could account for the reduced H_2_O_2_ emission observed in our study. Increased ONOO^–^ can result in increased protein nitration of tyrosine residues, potentially leading to altered protein function. Therefore, as an indirect marker of oxidative/nitrosative stress, we measured the effect of NITR on levels of nitrotyrosine via immunohistochemical staining of TA muscles. *Mdx* muscles had significantly higher nitrotyrosine staining compared with CON muscles (*p* < 0.0001) and NITR increased nitrotyrosine staining in both CON (*p* < 0.05) and *mdx* (*p* < 0.0001) TA (Fig. 7a and Fig. S2). Importantly, NITR induced a dramatically greater increase in nitrotyrosine production in *mdx* muscles (2775% increase *vs* 82% increase in CON). Additionally, NITR further increased the presence of DAPI-stained nuclei in NITR supplemented *mdx* TA (*p* < 0.0001; Fig. 7b  and Fig. S2). To assess if the increased nitrotyrosine staining was associated with increased inflammation, we measured CD45^+^ immune cell infiltration and IgG via immunolabeling. In *mdx* TA, both CD45^+^ and IgG^+^ area was elevated compared with CON muscles (*p* < 0.001 and *p* < 0.01, respectively; Fig. 7c, d, respectively and Fig. S3). In contrast to nitrotyrosine staining, NITR had no effect on the CD45^+^ and IgG^+^ area in either strain (*p* > 0.05).

### Effect of NITR Supplementation on Muscle Architecture

**Fig. 8:**
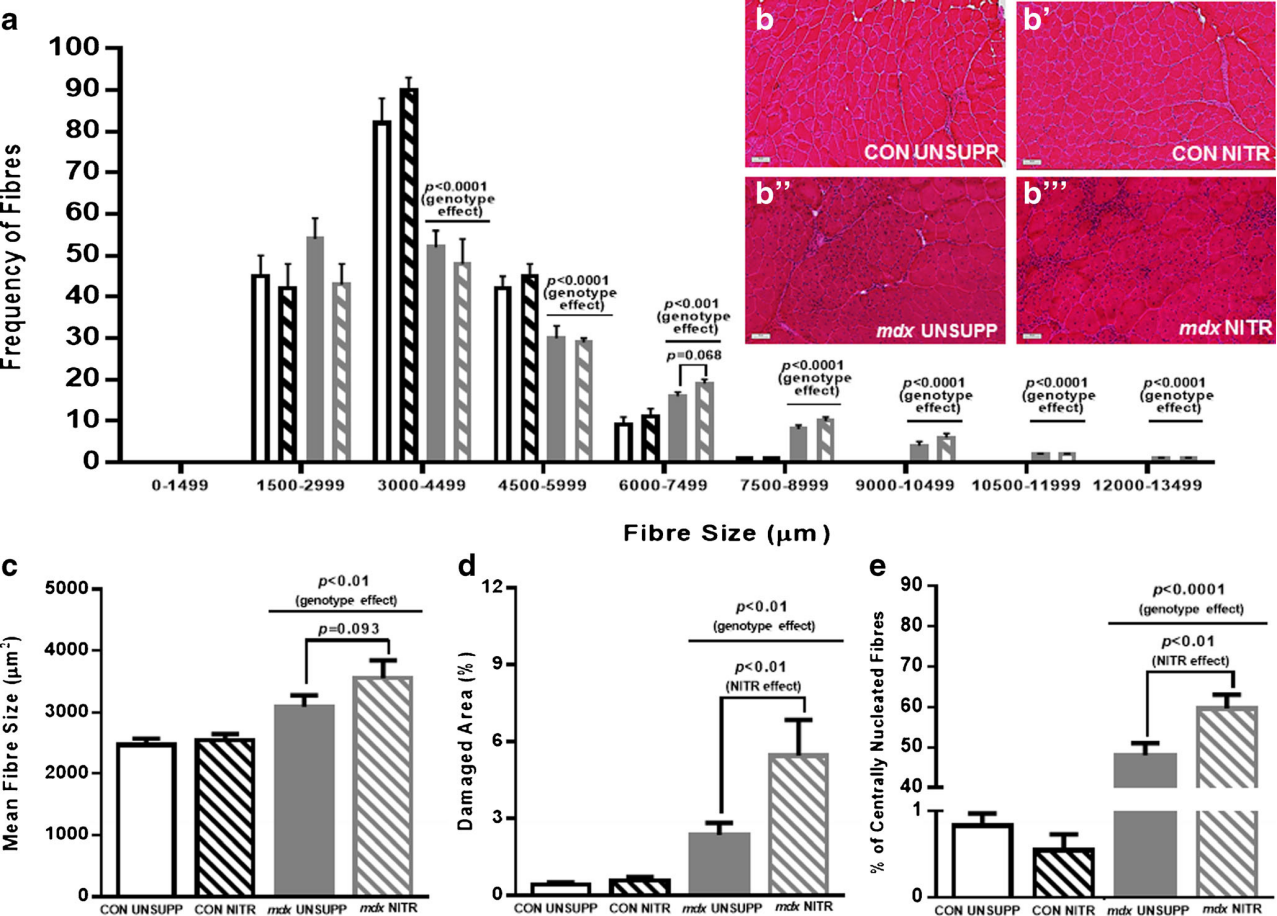
Histologic analysis of tibilalis anterior (TA) from unsupplemented (UNSUPP) and nitrate (NITR)-supplemented control (CON) and *mdx* mice. (**a**) The frequency histogram indicates an increase in fiber size of *mdx* TA with fibers more frequent from 6000 to 12,000 μm (*p* < 0.0001). NITR had no effect on the distribution of CON or *mdx* fibers, but there was a trend for an increased number of fibers around 6000 μm (*p* = 0.068). (**c**) Mean fiber size was significantly greater in *mdx* TA (*p* < 0.01) with a trend for NITR to increase fiber size in *mdx* TA (*p* = 0.093). (**d**) Damaged area and (**e**) percent centronucleated fibers was significantly higher in *mdx* TA (*p* < 0.01 and *p* < 0.0001, respectively) with NITR stimulating further damage and regeneration (*p* < 0.01). CON UNSUPP *n* = 11; CON NITR *n* = 12; *mdx* UNSUPP *n* = 11; *mdx* NITR *n* = 10

Finally, we assessed the effect of NITR on muscle fiber histopathology. As expected, intact *mdx* muscle fibers were significantly larger than fibers from CON muscles (Fig. 8a, c) which is representative of pseudohypertrophy, a hallmark histopathological feature of dystrophin-deficient muscle. Interestingly, there was a strong trend for NITR supplementation to increase the number of fibers between 6000 and 7499 μm^2^ (*p* = 0.068; Fig. 8a) and increase total mean fiber size (*p* = 0.093; Fig. 8c). The area of damage, as indicated by areas of inflammatory cell/nuclei infiltration, was significantly higher in *mdx* (*p* < 0.01; Fig. 8d) compared with CON TA sections, and NITR significantly increased the damage area in *mdx* muscle (*p* < 0.01). Centronucleated fibers, a marker of muscle cell regeneration, were significantly higher in *mdx* muscle (*p* < 0.0001; Fig. 8e) with NITR further increasing regeneration area in *mdx* sections (*p* < 0.01). These results show that NITR supplementation not only enhances muscle damage, but also regeneration in *mdx* TA but not in CON, which seems reflective of the increased ONOO^–^ production.

## Discussion

This is the first study to date to investigate NITR supplementation as a potential therapy for DMD, and we show that the metabolic perturbations in dystrophin-deficient skeletal muscle could not be overcome by enhancing nNOS-independent NO production. Instead, our data suggest that chronically increasing NO bioavailability without restoring nNOS protein expression and its regulatory role on metabolism, in fact, promotes pathological muscle damage, potentially via a ONOO^–^-dependent mechanism.

In the first instance, we investigated if impaired macronutrient uptake may be a contributing factor to the mitochondrial dysfunction in dystrophic muscle, as compromised transport of substrates across the sarcolemma could be a consequence of the loss of dystrophin and nNOS from the membrane. Specifically, we have investigated GU, as it is well established that GU during rest and contraction is regulated by NO [46]. The secondary loss of dystrophin-associated nNOS was confirmed in *mdx* TA via immunolabeling. Concurrently, we have demonstrated that both basal- and contraction-induced GU in both *mdx* UNSUPP EDL and SOL is comparable with CON. We have demonstrated in our study that NITR increases contraction-induced GU in CON EDL but has no effect in CON SOL. Indeed, we have shown previously that there are greater effects of NOS inhibition on EDL than SOL [46], likely because of a greater comparative nNOS expression in fast-twitch *versus* slow-twitch muscles [46, 58] and the higher antioxidant enzymes in slow-twitch muscles which may buffer the effects of NO [59]. In both CON EDL and SOL muscles, however, NITR did not affect basal GU rate. This could infer that the NITR dosage administered in our study is sufficient to modulate non-cyclic guanosine monophosphate (cGMP)-dependent contraction-induced glucose transporter 4-mediated GU [60] but perhaps not cGMP-dependent glucose transporter 1 basal [61] events. A notable limitation of our study is that we did not quantify cGMP levels in EDL and SOL muscles. However, in light of a recent study which demonstrated that even low-dose (0.35 mM) NITR therapy for ~2 weeks (in comparison with the 1 mM NITR dosage for 8 weeks administered in our study) was sufficient to induce ~3-fold increases in cGMP levels in rat skeletal muscle, this seems unlikely. Rather, basal GU is likely regulated in the first instance by glucose utilization, thus increasing NO signaling without the normal simultaneous increase in muscle work (and thus glucose utilization) results in an unchanged basal GU. Unexpectedly and in contrast to CON EDL, NITR reduced basal GU in *mdx* EDL and SOL. Taken with the fact that NITR stimulated contraction-induced GU in CON but further depressed it in *mdx* muscle, our data suggest that NITR-derived NO is being diverted away from its biomodulatory effects on GU. Presumably, this is because in *mdx* muscle, in which O_2_^–^ production is notoriously increased [57], NITR-generated NO is being sequestered into ONOO^–^ production instead of cGMP activation, thus reducing the proportional NO available to GU signaling despite an increased NITR–nitrite–NO pool.

We have assessed various indices of mitochondrial respiratory function in permeabilized RG and WG gastrocnemius fiber bundles. Permeabilization of intact muscle bundles and delivery of optimal substrate concentrations allows for the measurement of the mitochondrial capacity independent of substrate delivery capacity. Indeed, even in this optimized environment, we demonstrate a reduced capacity (up to 25% of CON) to phosphorylate ADP in both WG and RG from the *mdx* mouse. This is consistent with others [62, 63] who have reported similar depressions in ADP-stimulated phosphorylating respiration in *mdx* skeletal muscle fibers. NITR did not improve phosphorylating or maximal uncoupled respiration in either CON or *mdx* skeletal muscle but did decrease CIV activity in both CON and *mdx* RG. CIV inhibition is an established effect of reversible competitive binding of NO to heme-copper sites *in lieu* of O_2_ on CIV, in addition to CI and CIII [64–66]. Despite the inhibitory effect of NITR on CIV activity and therefore ETS respiratory capacity, the lack of effect on phosphorylating and maximal uncoupled respiration was unexpected, as NITR has been previously shown to improve various mitochondrial properties through stimulation of mitochondrial biogenesis and improved coupling of O_2_ consumption to ATP production [37]. As NO is a highly reactive molecule that, to exert its biological role, must be produced in close proximity to its effector targets, the exogenous NO source afforded by NITR supplementation in our study may not be penetrating the muscle fibers sufficiently, or in sufficient concentration, to modulate mitochondrial function. This is particularly true of the mitochondrial function governed by nuclear gene regulation such as mitochondrial biogenesis and uncoupling. Aquilano et al. [67], for instance, have demonstrated that the loss of nNOS-generated NO production nearby the nucleus is a causative factor of the impairment of mitochondrial biogenesis in skeletal muscle. Thus, while we have evidence of NITR-derived NO penetrating the mitochondria to induce regulatory adaptations such as inhibition of CIV activity, overall respiratory capacity which is dictated predominantly by mitochondrial density and coupling is seemingly unaffected, even in CON mice. This is likely due to the chronic supplementation period and particular dosage employed in our study. For example, similar to our study, Hezel et al. [68] did not observe any changes in mitochondrial parameters following 17 months of NITR supplementation in healthy mice. In contrast, others have shown beneficial mitochondrial modulation following much shorter supplementation periods [37, 69]. Ashmore et al. [38] have recently demonstrated that NITR dosage is also important to the control of the nuclear signaling of mitochondrial biogenesis in which low- (0.35 mM), medium- (0.7 mM), and high- (1.4 mM) dose NITR therapy (for 15–18 days) in rats had differential effects on peroxisome proliferator-activated receptor α/β/δ signaling, PGC-1α expression, CS activity, and mitochondrial fatty acid oxidation. These data highlight that the promotion of mitochondrial biogenesis might be an acute, dose-specific response to shorter-term increases in skeletal muscle NO signaling, which may switch off or become desensitized in response to more chronic, prolonged increases in NO production.

The reduced capacity for *mdx* skeletal muscle to phosphorylate ADP and to ramp up respiration during times of metabolic stress may be reflective of uncoupled respiration. In our study, state 4 respiration was significantly less in both WG and RG of *mdx* mice and the RCR was lower in *mdx* RG respiring on CI + II substrates highlighting that respiratory control is compromised in the muscle that is most dependent upon mitochondrial oxidative ATP production (i.e. red oxidative muscle). When considered in context of a depressed state 3 and 4 respiration, tighter respiratory control would be required to maintain the ΔΨ and drive for ATP synthesis, especially given the heightened energy requirements of dystrophic muscle. Indeed, our observations of a depolarized ΔΨ in isolated *mdx* mitochondria (C.A. Timpani, A. Hayes and E. Rybalka, unpublished observations) indicate insufficient coupling to maintain the drive for ATP synthesis in red muscle at least. Intriguingly, NITR decreased the RCR only in *mdx* muscles, indicative of mitochondrial uncoupling. Uncoupling may be a beneficial adaptation to ETS dysfunction, to prevent potential hyperpolarization of the ΔΨ which is an initiator of mitochondria-mediated apoptosis [70]. Certainly, the role of NITR-derived NO in the regulation of mitochondrial coupling efficiency is unclear as some studies have demonstrated an enhanced coupling efficiency of human skeletal muscle [37], while others have shown a reduced coupling efficiency of rodent skeletal muscle [38]. Despite the obvious species differences between these studies, these data highlight that NITR-derived NO has a modulatory role on the expression of uncoupling protein 3 (through increased peroxisome proliferator-activated receptor-α activation [38]) and adenine nucleotide translocase expression, and seemingly regulates the leakiness of several respiratory complexes—all of which contribute to the coupled state of skeletal muscle mitochondria. However, this role requires further elucidation.

A reduced mitochondrial pool (particularly viable mitochondria) could also explain the decreased OXPHOS capacity of dystrophic skeletal muscle in our study. We saw no genotype or muscle-specific differences in CS activity (a marker of mitochondrial density) in our study; however, we did see differential expression of ETC complex proteins in *mdx* RG and WG whereby complex proteins generally decreased in RG but increased in WG. This suggests a reduced respiratory capacity despite increased/unchanged mitochondrial density in *mdx* RG, in particular. We [23] and others [22, 62, 71] have previously reported this, highlighting that a reduced mitochondrial functional and/or physical density does not account for the decreased mitochondrial respiration associated with dystrophin-deficiency, but rather that the mitochondrial pool is intrinsically defective. While NITR had no effect on complex expression in RG from either strain, most complexes (except CIV), were upregulated in NITR-supplemented CON but not NITR-supplemented *mdx* WG. In fact, the only observed effect of NITR in WG that was consistent across strains was an increased CS activity, and this was reproducible in the RG from *mdx* but not CON mice. Our finding is curious as Ashmore et al. [38] demonstrated that a high (1.4 mM) NITR diet increases CS activity in red SOL muscle from healthy rats, although a low (0.35 mM) and medium (0.7 mM) diet did not. In context, we supplemented our mice with 1 mM NITR. Our data thus suggest that there are variations in the response of different fiber types to NITR-derived NO dosages, in which type II fibers are more responsive to a lower NO concentration. Irrespective of this, changes in mitochondrial CS activity and electron transport chain complex expression induced by NITR did not translate to improved mitochondrial respiration in either CON or *mdx* muscles in our study.

We found in various respiratory states that NITR reduced H_2_O_2_ production in *mdx* but not CON skeletal muscle. This would immediately seem to be beneficial, as ROS production is elevated in dystrophic muscle [57] and NO reduces oxidative stress at the level of the ETS [72]. However, excessive NO can lead to the generation of RNS in the presence of O_2_^–^. In addition to the inhibition of CIV, NO inhibits electron transfer at CI and CIII of the ETS [73], producing O_2_^–^ anions that interact with NO to produce ONOO^–^ which can induce cellular damage [74]. In our study, we have demonstrated elevated nitrotyrosine content in *mdx* TA muscles, which is consistent with increased ONOO^–^ production, and this was dramatically exacerbated by NITR (2775% increase). Nitrotyrosine labeling corresponded with an increased area of damage in NITR-supplemented *mdx* TA sections. In previous studies, NO donor therapy has been shown to reduce the area of damage in dystrophic muscle, but as NO donors are typically given in combination with anti-inflammatories [29, 33], our data suggest that the anti-inflammatory component of these co-compounds is perhaps the more pertinent effector. NITR also increased the proportion of centronucleated fibers in *mdx* muscles, which has been previously observed with NO donors [29, 30] and is reflective of an enhanced regenerative capacity in response to NITR-induced damage. NO is a known stimulator of satellite cell proliferation, which is crucial to skeletal muscle regeneration following damage [75] and is notably defective in dystrophin-deficient muscle [76, 77]. As dystrophic muscle is in a state of enhanced oxidative stress superfluous NITR-derived NO bioavailability appears detrimental to dystrophic muscle by promoting excess ONOO^–^ formation which, in turn, may exceed antioxidant buffering capacity to promote muscle damage and escalate pathology. This effect may be more evident in predominantly white fast-twitch glycolytic muscles (such as TA) owing to the lower endogenous antioxidant content and therefore NO handling capacity; however, further investigation is required to elucidate if this is true. It is also possible that the absence of nNOS protein expression, its translocational capacity to deliver NO to specific intracellular sites, and the metabolic modulatory effects it exerts may account for the deleterious effect that NITR had on dystrophic muscle histopathology in our study, as breeding transgenic overexpressing nNOS mice with the *mdx* strain results in significant improvements to dystrophic muscle architecture [26, 27]. NITR therapy, however, might be beneficial for the stimulation of satellite cell replication and dystrophic skeletal muscle regeneration as we observed elevated presence of DAPI-positive nuclei in NITR-treated *mdx* TA. While we did not stain for Pax-7 (a satellite cell marker), we did label CD45^+^ immune cell infiltrate and IgG deposition within the muscle cross-sections—neither of these measures were affected by NITR SUPP, suggesting that the NITR-dependent increase in nuclei content is most likely reflective of an enhanced satellite cell pool. Therefore, NITR therapy could be beneficial especially if mitochondrial O_2_^–^ production could be pharmacologically attenuated and RNS-induced damage prevented (such as with antioxidant therapy).

In summary, our study is the first to demonstrate that an 8-week supplementation regimen of NITR in drinking water cannot overcome the metabolic dysfunction observed in the *mdx* mouse model of DMD. We are the first to examine contraction-induced GU in the *mdx* model and to demonstrate that NITR supplementation reduces otherwise normal GU in *mdx* muscles and cannot positively modulate mitochondrial function. Although NITR supplementation reduced mitochondrial H_2_O_2_ emission, it induced mitochondrial uncoupling in RG, increased muscle fiber nitrosylation (and therefore ONOO^–^ radicals), and promoted skeletal muscle damage. Our data are consistent with recent literature linking NO to muscle soreness [78]. Together this suggests that enhancing endogenous NO production via exogenous NITR therapy is contraindicative for the treatment of DMD. This is potentially owing to the fact that there is no concomitant increase in nNOS protein expression and its regulatory role over metabolic flux control, and that excessive ROS promotes RNS production which actually reduced NO bioavailability.

There were some limitations to our study that are worthy of mention. In the first instance, we did not quantify cGMP levels in EDL and SOL muscles, and thus cannot confirm that in the presence of heightened O_2_^–^ production, NO is diverted away from intracellular signaling pathways (i.e., cGMP production, nucleus signaling of mitochondrial biogenesis) and into RNS formation. This was because whole EDL and SOL was required for our primary measure being radioactive GU, and that our other tissues were not immediately snap frozen, thus cGMP was heavily degraded beyond detectable levels. Second, while simultaneous measurement of O_2_ flux and H_2_O_2_ emission has previously been well characterized and reported [50–52], a potential limitation of this assay is that the supraphysiological chamber pO_2_ used to overcome O_2_ diffusion limitations of permeabilized muscle fiber preparations, may lead to nonphysiological rates of H_2_O_2_ emission [79]. Therefore, it should be acknowledged that the H_2_O_2_ measured using the present assay may not completely recapitulate *in vivo* mitochondrial ROS emission rates.

In conclusion, our data are in stark contrast to previous findings of significant improvements in the dystrophic condition following NO donor therapy, and in patients with Becker muscular dystrophy following nitrite supplementation, suggesting that long-term NITR/NO supplementation requires better characterization, particularly in conditions of heightened oxidative and/or metabolic stress such as in DMD. While the precise myopathological mechanisms of NITR has not been fully elucidated in the present study, our data are of particular importance considering NITR therapy is currently in clinical trials for the treatment of patients with DMD.

## Electronic supplementary material

Below is the link to the electronic supplementary material.

ESM 1 (PDF 516 kb)

Fig. S1 Proportion of dystrophin and neuronal nitric oxide synthase (nNOS) immunoreactivity in unsupplemented and supplemented control and *mdx* tibilalis anterior (JPG 160 kb)

Fig. S2 Immunohistological nitrotyrosine analysis of tibilalis anterior from unsupplemented and nitrate (NITR)-supplemented control (CON) and *mdx* mice (GIF 609 kb)

High resolution image (TIF 993 kb)

Fig. S3 Immunohistologic analysis of CD45 infiltration and IgG staining in tibilalis anterior from unsupplemented and nitrate (NITR)-supplemented control (CON) and *mdx* mice (GIF 420 kb)

High resolution image (TIF 719 kb)

## References

[1] Monaco AP, Bertelson CJ, Middlesworth W (1985). Detection of deletions spanning the Duchenne muscular dystrophy locus using a tightly linked DNA segment. Nature.

[2] Emery A (1991). Population frequencies of inherited neuromuscular diseases—a world survey. Neuromuscul Disord.

[3] Hoffman EP, Brown RH, Kunkel LM (1987). Dystrophin: the protein product of the Duchenne muscular dystrophy locus. Cell.

[4] Heslop L, Morgan JE, Partridge TA (2000). Evidence for a myogenic stem cell that is exhausted in dystrophic muscle. J Cell Sci.

[5] Eagle M, Baudouin SV, Chandler C, Giddings DR, Bullock R, Bushby K (2002). Survival in Duchenne muscular dystrophy: improvements in life expectancy since 1967 and the impact of home nocturnal ventilation. Neuromuscul Disord.

[6] Timpani CA, Hayes A, Rybalka E (2015). Revisiting the dystrophin-ATP connection: how half a century of research still implicates mitochondrial dysfunction in Duchenne Muscular Dystrophy aetiology. Med Hypotheses.

[7] Ohlendieck K, Campbell KP (1991). Dystrophin-associated proteins are greatly reduced in skeletal muscle from mdx mice. J Cell Biol.

[8] Brenman JE, Chao DS, Xia H, Aldape K, Bredt DS (1995). Nitric oxide synthase complexed with dystrophin and absent from skeletal muscle sarcolemma in Duchenne muscular dystrophy. Cell.

[9] Chang WJ, Iannaccone ST, Lau KS (1996). Neuronal nitric oxide synthase and dystrophin-deficient muscular dystrophy. Proc Natl Acad Sci U S A.

[10] McConell GK, Rattigan S, Lee-Young RS, Wadley GD, Merry TL (2012). Skeletal muscle nitric oxide signaling and exercise: a focus on glucose metabolism. Am J Physiol Endocrinol Metab.

[11] Leary SC, Battersby BJ, Hansford RG, Moyes CD (1998). Interactions between bioenergetics and mitochondrial biogenesis. Biochim Biophys Acta.

[12] Thomas GD, Sander M, Lau KS, Huang PL, Stull JT, Victor RG (1998). Impaired metabolic modulation of α-adrenergic vasoconstriction in dystrophin-deficient skeletal muscle. Proc Natl Acad Sci U S A.

[13] Vaghy PL, Fang J, Wu W, Vaghy LP (1998). Increased caveolin-3 levels in mdx mouse muscles. FEBS Lett.

[14] Judge LM, Haraguchiln M, Chamberlain JS (2006). Dissecting the signaling and mechanical functions of the dystrophin-glycoprotein complex. J Cell Sci.

[15] Kameya S, Miyagoe Y, Nonaka I (1999). α1-syntrophin gene disruption results in the absence of neuronal-type nitric-oxide synthase at the sarcolemma but does not induce muscle degeneration. J Biol Chem.

[16] Li D, Yue Y, Lai Y, Hakim CH, Duan D (2011). Nitrosative stress elicited by nNOSμ delocalization inhibits muscle force in dystrophin‐null mice. J Pathol.

[17] Gücüyener K, Ergenekon E, Erbas D, Pinarli G, Serdaroğlu A (2000). The serum nitric oxide levels in patients with Duchenne muscular dystrophy. Brain Develop.

[18] Kasai T, Abeyama K, Hashiguchi T, Fukunaga H, Osame M, Maruyama K (2004). Decreased total nitric oxide production in patients with Duchenne muscular dystrophy. J Biomed Sci.

[19] Barton ER, Morris L, Kawana M, Bish LT, Toursel T (2005). Systemic administration of L-arginine benefits mdx skeletal muscle function. Muscle Nerve.

[20] Wehling-Henricks M, Oltmann M, Rinaldi C, Myung KH, Tidball JG (2009). Loss of positive allosteric interactions between neuronal nitric oxide synthase and phosphofructokinase contributes to defects in glycolysis and increased fatigability in muscular dystrophy. Hum Mol Genet.

[21] Vignos PJ, Lefkowitz M (1959). A biochemical study of certain skeletal muscle constituents in human progressive muscular dystrophy. J Clin Invest.

[22] Chi MMY, Hintz CS, McKee D (1987). Effect of Duchenne muscular dystrophy on enzymes of energy metabolism in individual muscle fibers. Metabolism.

[23] Rybalka E, Timpani CA, Cooke MB, Williams AD, Hayes A (2014). Defects in mitochondrial ATP synthesis in dystrophin-deficient mdx skeletal muscles may be caused by complex I insufficiency. PLOS ONE.

[24] Austin L, De Niese M, McGregor A, Arthur H, Gurusinghe A, Gould M (1992). Potential oxyradical damage and energy status in individual muscle fibres from degenerating muscle diseases. Neuromuscul Disord.

[25] Cole M, Rafael J, Taylor D, Lodi R, Davies K, Styles P (2002). A quantitative study of bioenergetics in skeletal muscle lacking utrophin and dystrophin. Neuromuscul Disord.

[26] Wehling M, Spencer MJ, Tidball JG (2001). A nitric oxide synthase transgene ameliorates muscular dystrophy in mdx mice. J Cell Biol.

[27] Tidball JG, Wehling-Henricks M (2004). Expression of a NOS transgene in dystrophin-deficient muscle reduces muscle membrane damage without increasing the expression of membrane-associated cytoskeletal proteins. Mol Genet Metab.

[28] Voisin V, Sebrie C, Matecki S (2005). L-arginine improves dystrophic phenotype in mdx mice. Neurobiol Dis.

[29] Brunelli S, Sciorati C, D'Antona G (2007). Nitric oxide release combined with nonsteroidal antiinflammatory activity prevents muscular dystrophy pathology and enhances stem cell therapy. Proc Natl Acad Sci U S A.

[30] Mizunoya W, Upadhaya R, Burczynski FJ, Wang G, Anderson JE (2011). Nitric oxide donors improve prednisone effects on muscular dystrophy in the mdx mouse diaphragm. Am J Physiol Cell Physiol.

[31] Vianello S, Consolaro F, Bich C (2014). Low doses of arginine butyrate derivatives improve dystrophic phenotype and restore membrane integrity in DMD models. FASEB J.

[32] Archer JD, Vargas CC, Anderson JE (2006). Persistent and improved functional gain in mdx dystrophic mice after treatment with L-arginine and deflazacort. FASEB J.

[33] Uaesoontrachoon K, Quinn JL, Tatem KS (2014). Long-term treatment with naproxcinod significantly improves skeletal and cardiac disease phenotype in the mdx mouse model of dystrophy. Hum Mol Genet.

[34] Thomas GD, Ye J, De Nardi C, Monopoli A, Ongini E, Victor RG (2012). Treatment with a nitric oxide-donating NSAID alleviates functional muscle ischemia in the mouse model of Duchenne muscular dystrophy. PLOS ONE.

[35] Anderson JE, Vargas C (2003). Correlated NOS-Iμ and myf5 expression by satellite cells in mdx mouse muscle regeneration during NOS manipulation and deflazacort treatment. Neuromuscul Disord.

[36] Lundberg JO, Weitzberg E, Gladwin MT (2008). The nitrate-nitrite-nitric oxide pathway in physiology and therapeutics. Nat Rev Drug Discov.

[37] Larsen FJ, Schiffer TA, Borniquel S (2011). Dietary inorganic nitrate improves mitochondrial efficiency in humans. Cell Metab.

[38] Ashmore T, Roberts LD, Morash AJ (2015). Nitrate enhances skeletal muscle fatty acid oxidation via a nitric oxide-cGMP-PPAR-mediated mechanism. BMC Biol.

[39] Bailey SJ, Winyard P, Vanhatalo A (2009). Dietary nitrate supplementation reduces the O2 cost of low-intensity exercise and enhances tolerance to high-intensity exercise in humans. J Appl Physiol.

[40] Larsen F, Weitzberg E, Lundberg J, Ekblom B (2007). Effects of dietary nitrate on oxygen cost during exercise. Acta Physiol.

[41] Bailey SJ, Fulford J, Vanhatalo A (2010). Dietary nitrate supplementation enhances muscle contractile efficiency during knee-extensor exercise in humans. J Appl Physiol.

[42] Kenjale AA, Ham KL, Stabler T (2011). Dietary nitrate supplementation enhances exercise performance in peripheral arterial disease. J Appl Physiol.

[43] Nelson MD, Rosenberry R, Barresi R (2015). Sodium nitrate alleviates functional muscle ischaemia in patients with Becker muscular dystrophy. J Physiol.

[44] Carlström M, Larsen FJ, Nyström T (2010). Dietary inorganic nitrate reverses features of metabolic syndrome in endothelial nitric oxide synthase-deficient mice. Proc Natl Acad Sci U S A.

[45] Hernández A, Schiffer TA, Ivarsson N (2012). Dietary nitrate increases tetanic [Ca2+] i and contractile force in mouse fast‐twitch muscle. J Physiol.

[46] Merry TL, Steinberg GR, Lynch GS, McConell GK (2010). Skeletal muscle glucose uptake during contraction is regulated by nitric oxide and ROS independently of AMPK. Am J Physiol Endocrinol Metab.

[47] Hong YH, Frugier T, Zhang X (2015). Glucose uptake during contraction in isolated skeletal muscles from neuronal nitric oxide synthase μ knockout mice. J Appl Physiol.

[48] Stephenson EJ, Stepto NK, Koch LG, Britton SL, Hawley JA (2012). Divergent skeletal muscle respiratory capacities in rats artificially selected for high and low running ability: a role for Nor1?. J Appl Physiol.

[49] Stephenson EJ, Camera DM, Jenkins TA (2012). Skeletal muscle respiratory capacity is enhanced in rats consuming an obesogenic Western diet. Am J Physiol Endocrinol Metab.

[50] Makrecka-Kuka M, Krumschnabel G, Gnaiger E (2015). High-resolution respirometry for simultaneous measurement of oxygen and hydrogen peroxide fluxes in permeabilized cells, tissue homogenate and isolated mitochondria. Biomolecules.

[51] Hickey AJ, Renshaw GM, Speers-Roesch B (2012). A radical approach to beating hypoxia: depressed free radical release from heart fibres of the hypoxia-tolerant epaulette shark (Hemiscyllum ocellatum). J Comp Physiol B.

[52] Krumschnabel G, Fontana-Ayoub M, Sumbalova Z (2015). Simultaneous high-resolution measurement of mitochondrial respiration and hydrogen peroxide production. Methods Mol Biol.

[53] Larsen S, Nielsen J, Hansen CN (2012). Biomarkers of mitochondrial content in skeletal muscle of healthy young human subjects. J Physiol.

[54] Vigelsø Hansen A, Andersen NB, Dela F (2014). The relationship between skeletal muscle mitochondrial citrate synthase activity and whole body oxygen uptake adaptations in response to exercise training. Int J Physiol Pathophysiol Pharmacol.

[55] Srere P (1969). 1] Citrate synthase:[EC 4.1. 3.7. Citrate oxaloacetate-lyase (CoA-acetylating). Methods Enzymol.

[56] Shavlakadze T, White J, Hoh JF, Rosenthal N, Grounds MD (2004). Targeted expression of insulin-like growth factor-I reduces early myofiber necrosis in dystrophic mdx mice. Mol Ther.

[57] Disatnik MH, Dhawan J, Yu Y (1998). Evidence of oxidative stress in *mdx* mouse muscle: studies of the pre-necrotic state. J Neurol Sci.

[58] Kobzik L, Reid MB, Bredt DS, Stamler JS (1994). Nitric oxide in skeletal muscle. Nature.

[59] Ji LL, Fu R, Mitchell EW (1992). Glutathione and antioxidant enzymes in skeletal muscle: effects of fiber type and exercise intensity. J Appl Physiol.

[60] Richter EA, Hargreaves M (2013). Exercise, GLUT4, and skeletal muscle glucose uptake. Physiol Rev.

[61] Ren J-M, Marshall B, Gulve E (1993). Evidence from transgenic mice that glucose transport is rate-limiting for glycogen deposition and glycolysis in skeletal muscle. J Biol Chem.

[62] Kuznetsov AV, Winkler K, Wiedemann F, von Bossanyi P, Dietzmann K, Kunz WS (1998). Impaired mitochondrial oxidative phosphorylation in skeletal muscle of the dystrophin-deficient mdx mouse. Mol Cell Biochem.

[63] Passaquin AC, Renard M, Kay L (2002). Creatine supplementation reduces skeletal muscle degeneration and enhances mitochondrial function in *mdx* mice. Neuromuscul Disord.

[64] Brown GC, Borutaite V, editors. Nitric oxide, cytochrome c and mitochondria. Biochemical Society Symposia; 1999: Portland Press Limited. 10.1042/bss0660017 10989653

[65] Brunori M, Giuffrè A, Forte E, Mastronicola D, Barone MC, Sarti P. Control of cytochrome c oxidase activity by nitric oxide. Biochimica et Biophysica Acta (BBA)-Bioenergetics 2004;1655:365-371. 10.1016/j.bbabio.2003.06.008 15100052

[66] Sarti P, Giuffrè A, Barone MC, Forte E, Mastronicola D, Brunori M (2003). Nitric oxide and cytochrome oxidase: reaction mechanisms from the enzyme to the cell. Free Radic Biol Med.

[67] Aquilano K, Baldelli S, Ciriolo MR (2014). Nuclear recruitment of neuronal nitric-oxide synthase by α-syntrophin is crucial for the induction of mitochondrial biogenesis. J Biol Chem.

[68] Hezel MP, Liu M, Schiffer TA (2015). Effects of long-term dietary nitrate supplementation in mice. Redox Biol.

[69] Nisoli E, Falcone S, Tonello C (2004). Mitochondrial biogenesis by NO yields functionally active mitochondria in mammals. Proc Natl Acad Sci U S A.

[70] Nagy G, Koncz A, Fernandez D, Perl A (2007). Nitric oxide, mitochondrial hyperpolarization, and T cell activation. Free Radic Biol Med.

[71] Jongpiputvanich S, Sueblinvong T, Norapucsunton T (2005). Mitochondrial respiratory chain dysfunction in various neuromuscular diseases. J Clin Neurosci.

[72] Cho H, Mu J, Kim JK (2001). Insulin resistance and a diabetes mellitus-like syndrome in mice lacking the protein kinase Akt2 (PKBβ). Science.

[73] Poderoso JJ, Carreras C, Lisdero C, Riobó N, Schöpfer F, Boveris A (1996). Nitric oxide inhibits electron transfer and increases superoxide radical production in rat heart mitochondria and submitochondrial particles. Arch Biochem Biophys.

[74] Beckman JS, Beckman TW, Chen J, Marshall PA, Freeman BA (1990). Apparent hydroxyl radical production by peroxynitrite: implications for endothelial injury from nitric oxide and superoxide. Proc Natl Acad Sci U S A.

[75] Anderson JE (2000). A role for nitric oxide in muscle repair: nitric oxide–mediated activation of muscle satellite cells. Mol Biol Cell.

[76] Biressi S, Miyabara EH, Gopinath SD, Carlig PM, Rando TA. A Wnt-TGFβ2 axis induces a fibrogenic program in muscle stem cells from dystrophic mice. Sci Transl Med 2014;6(267):267ra176. 10.1126/scitranslmed.3008411 PMC4350665 25520397

[77] Dumont NA, Wang YX, von Maltzahn J (2015). Dystrophin expression in muscle stem cells regulates their polarity and asymmetric division. Nat Med.

[78] Radak Z, Naito H, Taylor AW, Goto S (2012). Nitric oxide: Is it the cause of muscle soreness?. Nitric Oxide.

[79] Hoffman DL, Brookes PS (2009). Oxygen sensitivity of mitochondrial reactive oxygen species generation depends on metabolic conditions. J Biol Chem.

