# Supplementary figures and images for: Attempting to Compensate for Reduced Neuronal Nitric Oxide Synthase Protein with Nitrate Supplementation Cannot Overcome Metabolic Dysfunction but Rather Has Detrimental Effects in Dystrophin-Deficient mdx Muscle
Source: Neurotherapeutics. 2016 Dec 5;14(2):429–46. doi: 10.1007/s13311-016-0494-7 (PMC5398978; doi:10.1007/s13311-016-0494-7)

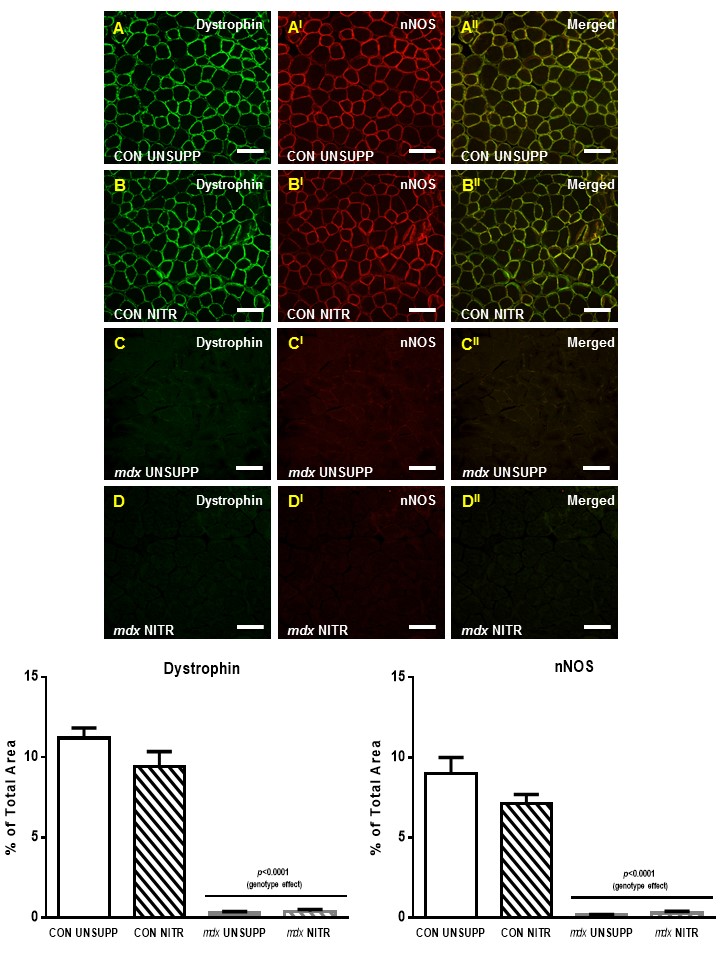

Supplement: Supplementary file 2 — Proportion of dystrophin and neuronal nitric oxide synthase (nNOS) immunoreactivity in unsupplemented and supplemented control and mdx tibilalis anterior (JPG 160 kb) [file 13311_2016_494_Fig9_ESM.jpg]

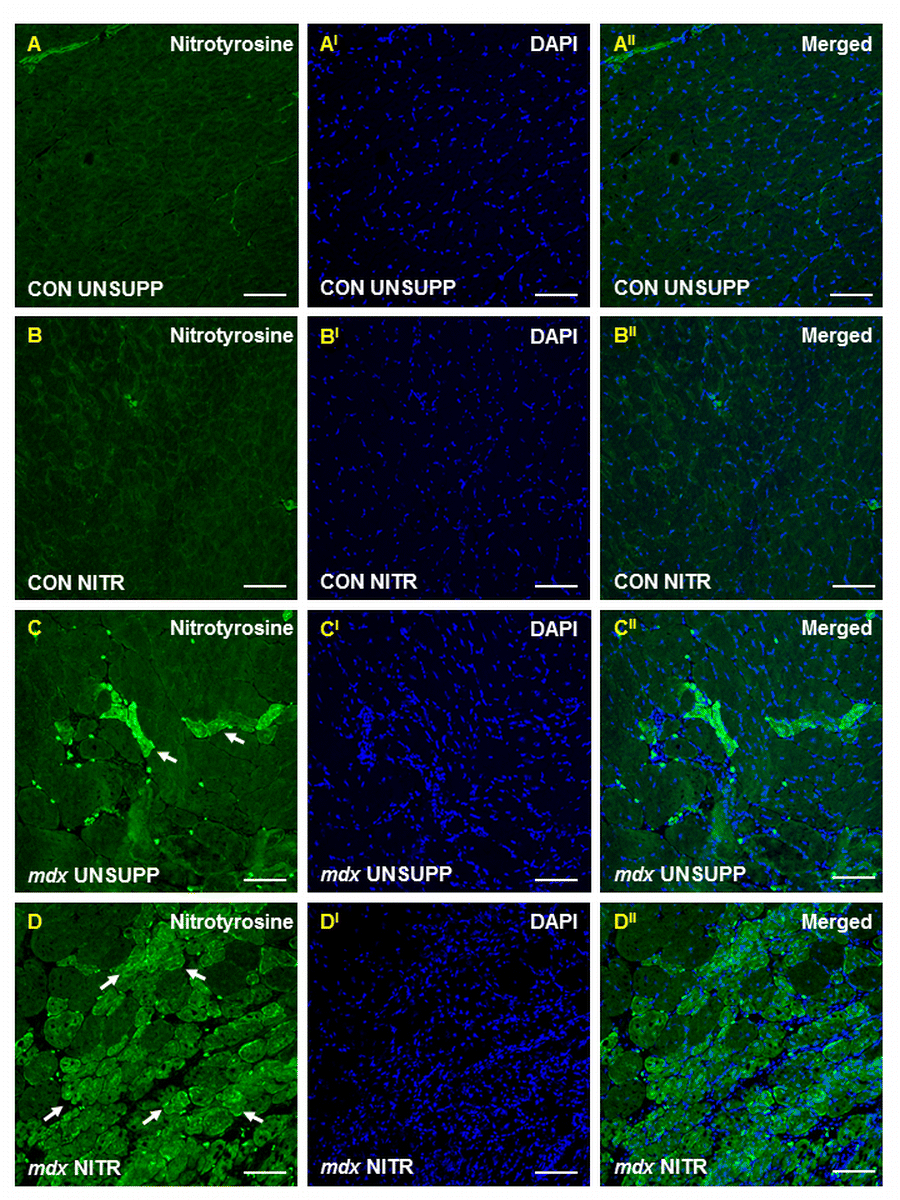

Supplement: Supplementary file 3 — Immunohistological nitrotyrosine analysis of tibilalis anterior from unsupplemented and nitrate (NITR)-supplemented control (CON) and mdx mice (GIF 609 kb) [file 13311_2016_494_Fig10_ESM.gif]

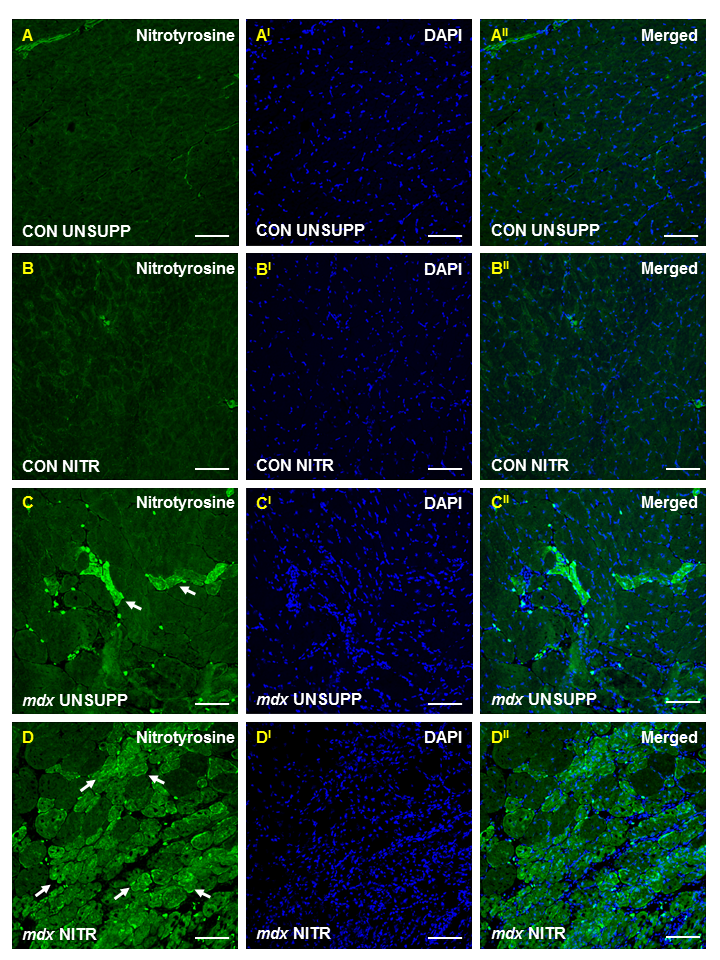

Supplement: Supplementary file 4 — High resolution image (TIF 993 kb) [file 13311_2016_494_MOESM2_ESM.tif]

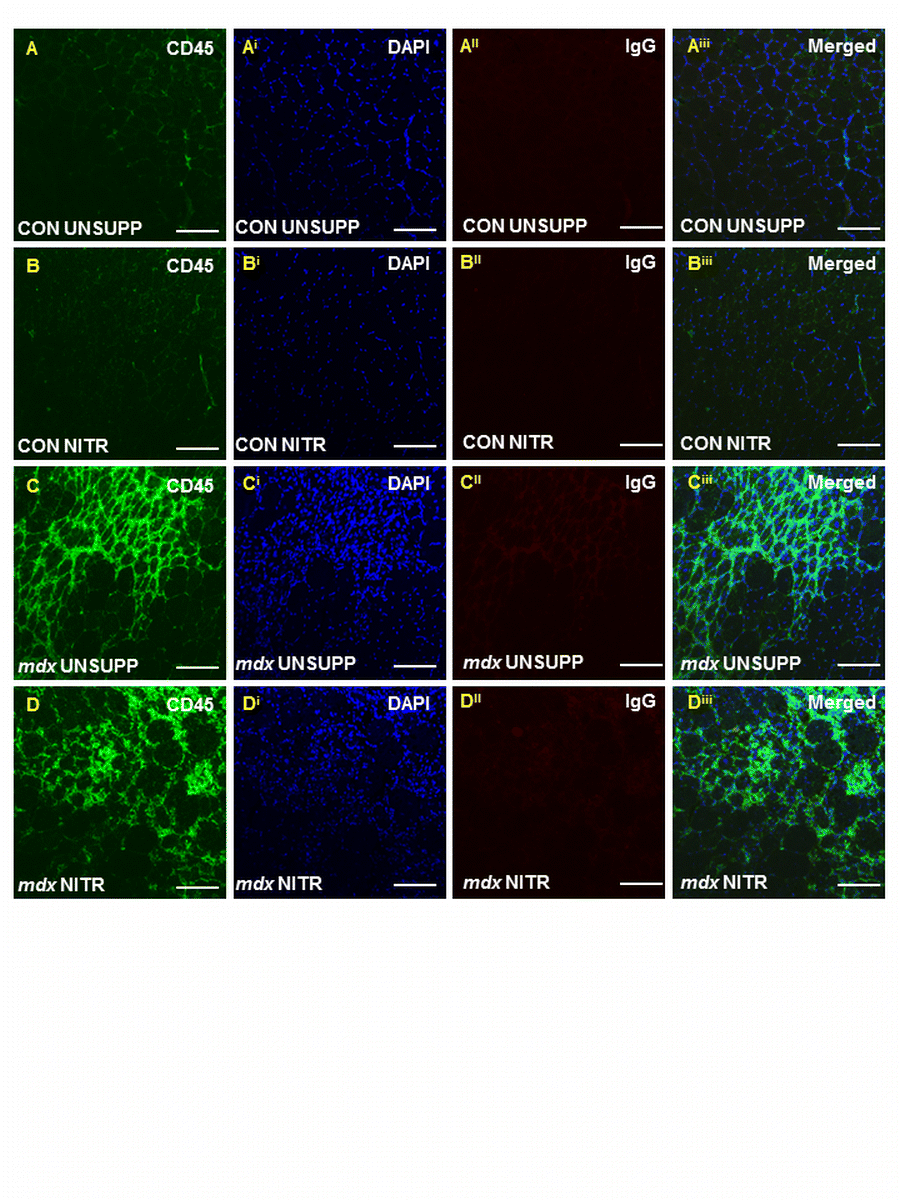

Supplement: Supplementary file 5 — Immunohistologic analysis of CD45 infiltration and IgG staining in tibilalis anterior from unsupplemented and nitrate (NITR)-supplemented control (CON) and mdx mice (GIF 420 kb) [file 13311_2016_494_Fig11_ESM.gif]

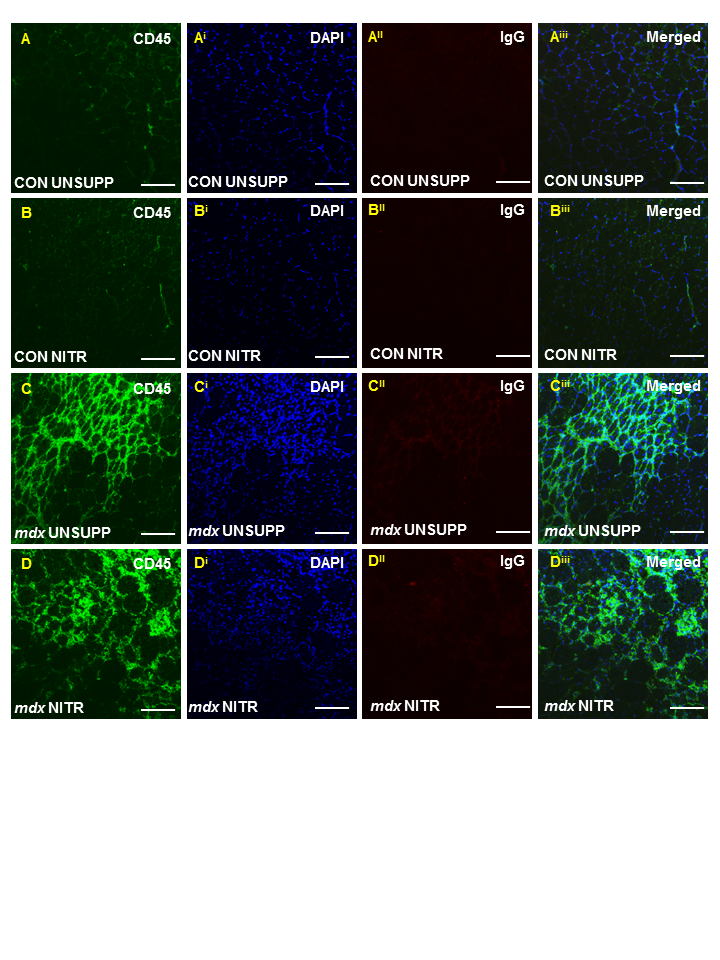

Supplement: Supplementary file 6 — High resolution image (TIF 719 kb) [file 13311_2016_494_MOESM3_ESM.tif]
